# Supplementary material for: Association between Sagittal Cervical Spinal Alignment and Degenerative Cervical Spondylosis: A Retrospective Study Using a New Scoring System
Source: J Clin Med. 2022 Mar 23;11(7):1772. doi: 10.3390/jcm11071772 (PMC8999493; doi:10.3390/jcm11071772)
Supplement: Supplementary file 1 [file jcm-11-01772-s001.zip › File_S1.pdf]

File S1: Sample images for DCS scoring.

Sample 1.

80/ male

C2-7 SVA: 9.2 mm

C2-7 ARA: 2.9°

	C23	C34	C45	C56	C67	Total score
Endplate sclerosis	0	2	1	2	1	6
Disc space narrowing	0	3	1	3	2	9
Anterior osteophyte	0	1	1	2	1	5
Posterior osteophyte	0	1	1	1	1	4
Listhesis	1	1	1	1	1	5
Facet joint	1	0	1	1	1	4
Total score	2	8	6	10	7	33

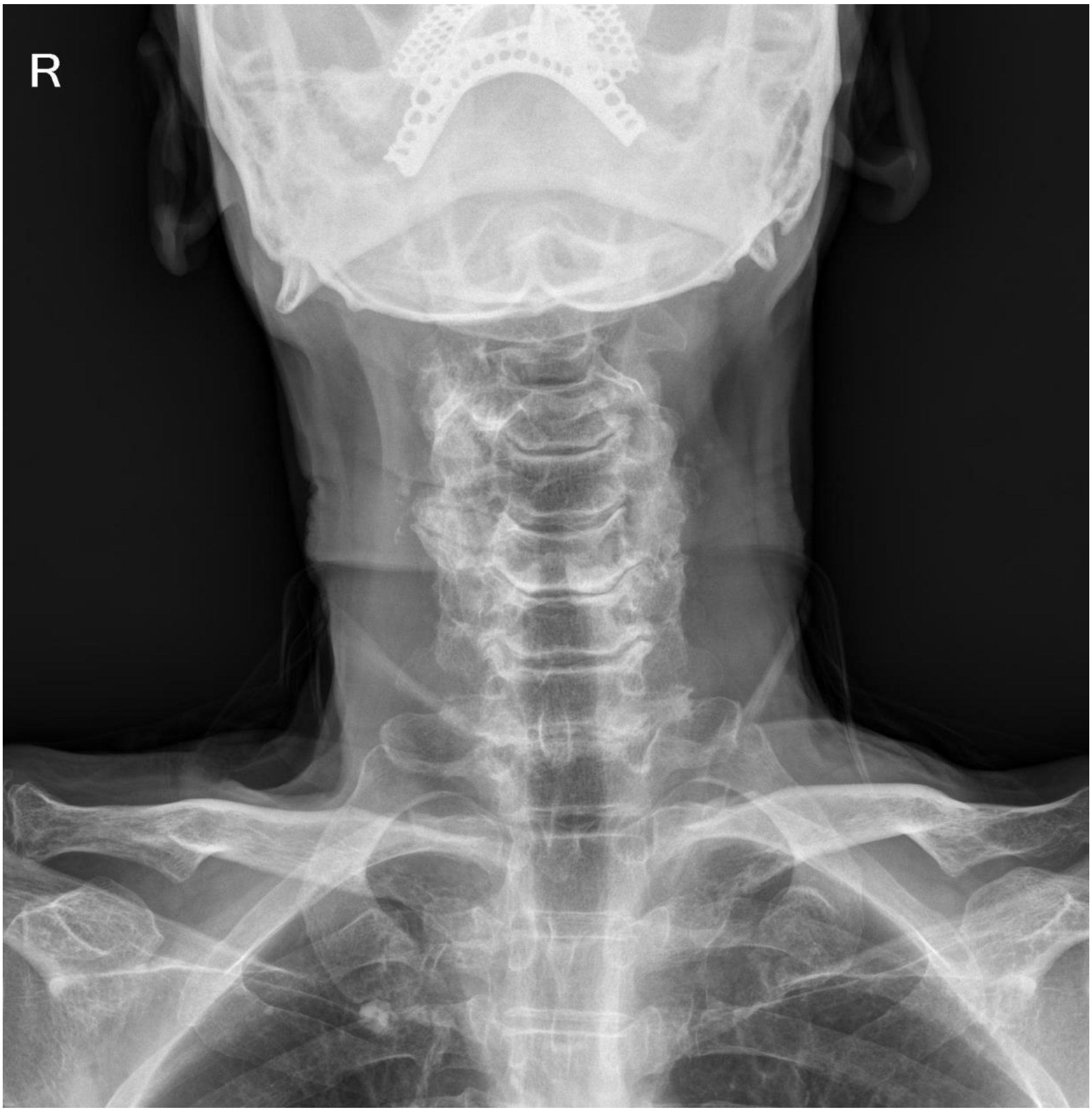

	C23	C34	C45	C56	C67	Total score
Endplate sclerosis	0	2	1	2	1	6
Disc space narrowing	0	3	1	3	2	9
Anterior osteophyte	0	1	1	2	1	5
Posterior osteophyte	0	1	1	1	1	4
Listhesis	1	1	1	1	1	5
Facet joint	1	0	1	1	1	4
Total score	2	8	6	10	7	33

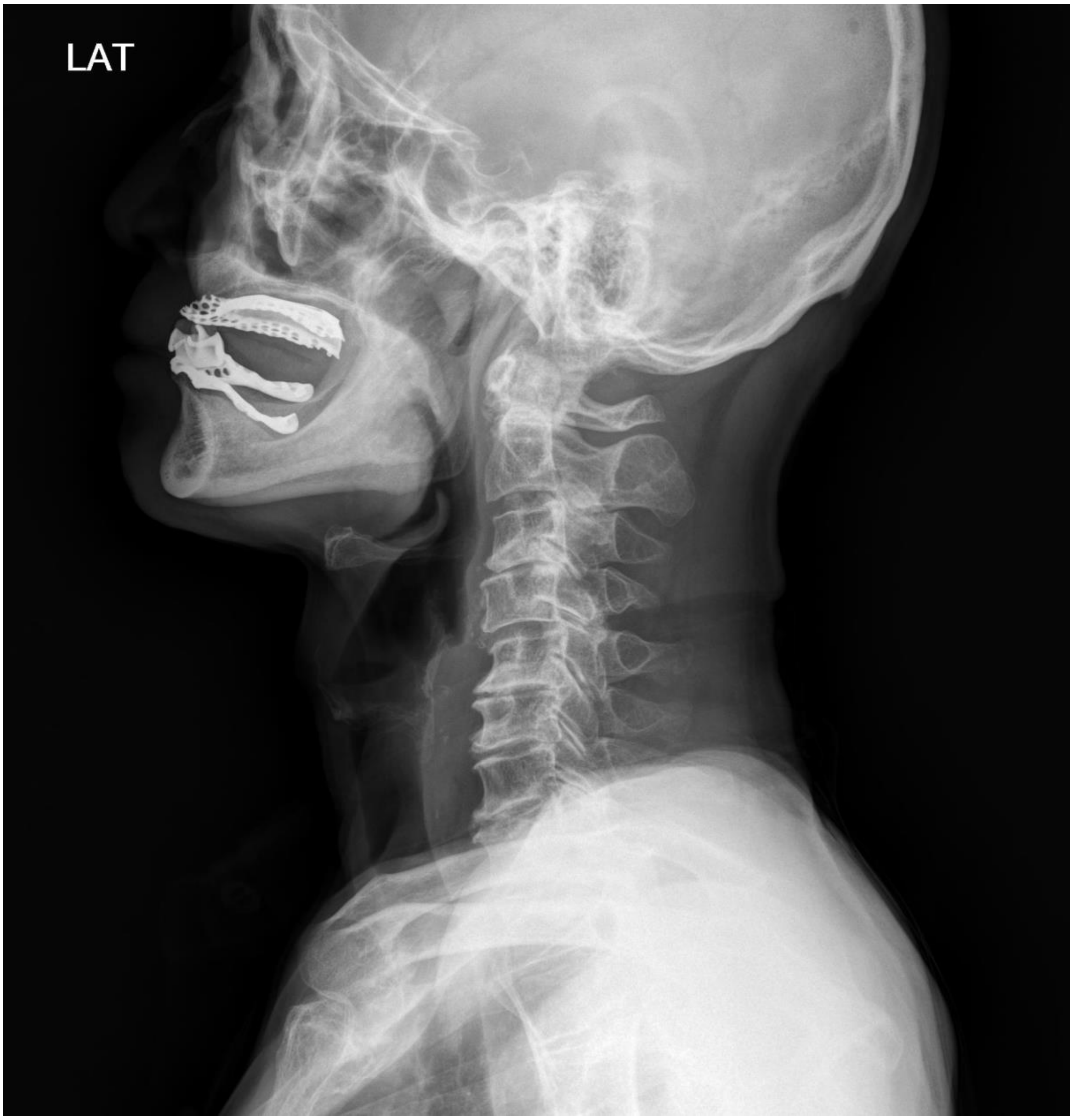

	C23	C34	C45	C56	C67	Total score
Endplate sclerosis	0	2	1	2	1	6
Disc space narrowing	0	3	1	3	2	9
Anterior osteophyte	0	1	1	2	1	5
Posterior osteophyte	0	1	1	1	1	4
Listhesis	1	1	1	1	1	5
Facet joint	1	0	1	1	1	4
Total score	2	8	6	10	7	33

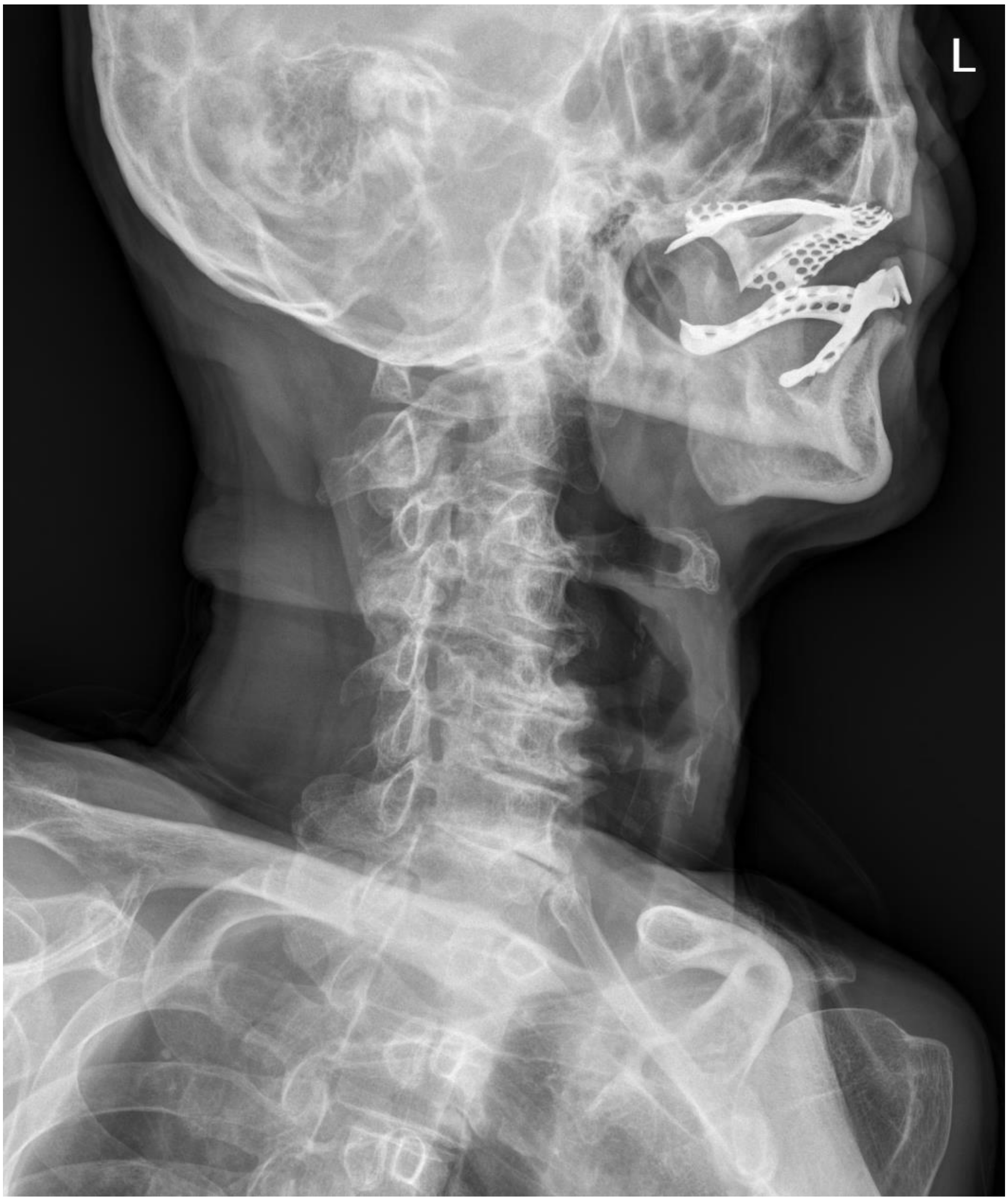

	C23	C34	C45	C56	C67	Total score
Endplate sclerosis	0	2	1	2	1	6
Disc space narrowing	0	3	1	3	2	9
Anterior osteophyte	0	1	1	2	1	5
Posterior osteophyte	0	1	1	1	1	4
Listhesis	1	1	1	1	1	5
Facet joint	1	0	1	1	1	4
Total score	2	8	6	10	7	33

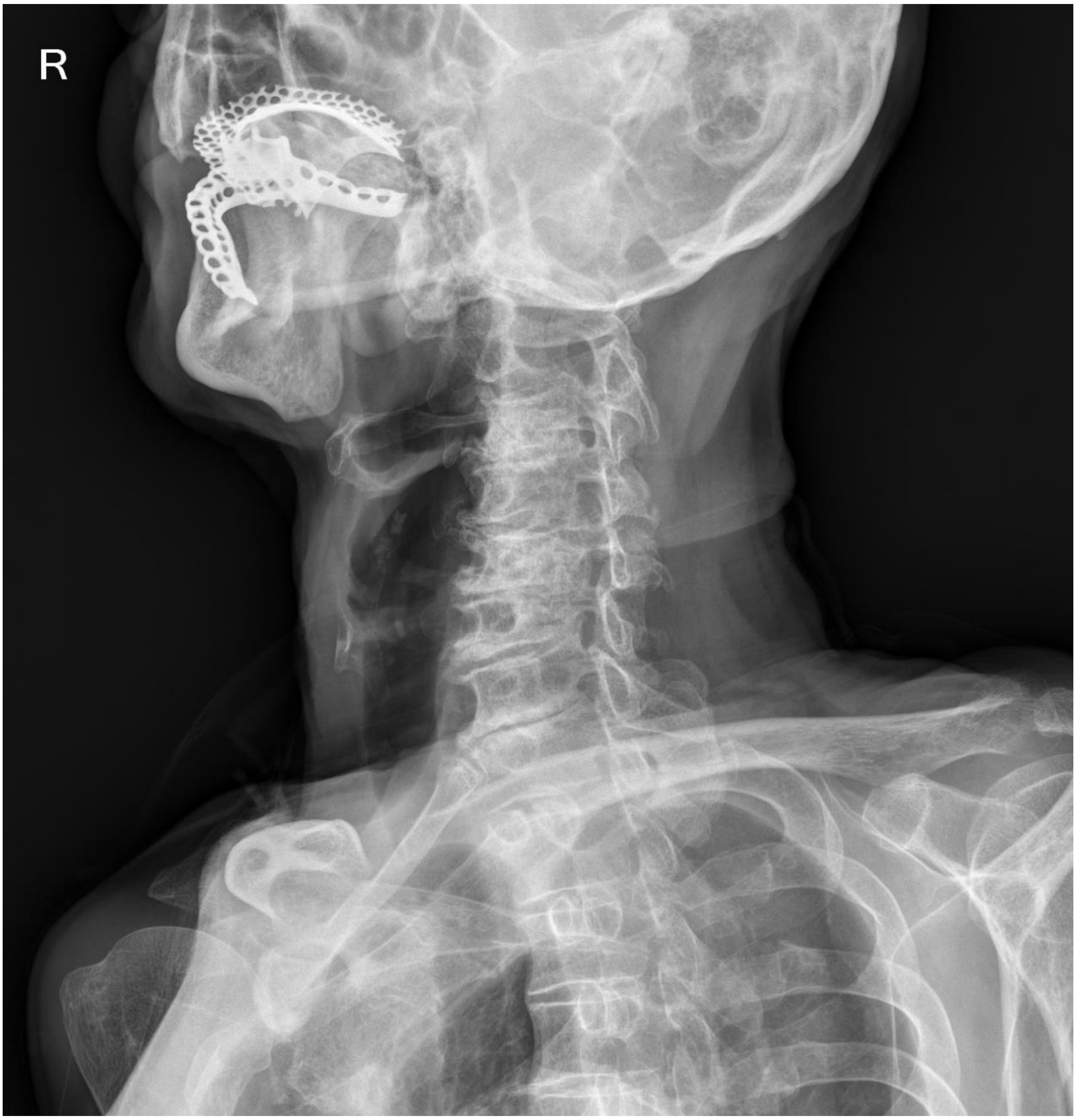

	C23	C34	C45	C56	C67	Total score
Endplate sclerosis	0	2	1	2	1	6
Disc space narrowing	0	3	1	3	2	9
Anterior osteophyte	0	1	1	2	1	5
Posterior osteophyte	0	1	1	1	1	4
Listhesis	1	1	1	1	1	5
Facet joint	1	0	1	1	1	4
Total score	2	8	6	10	7	33

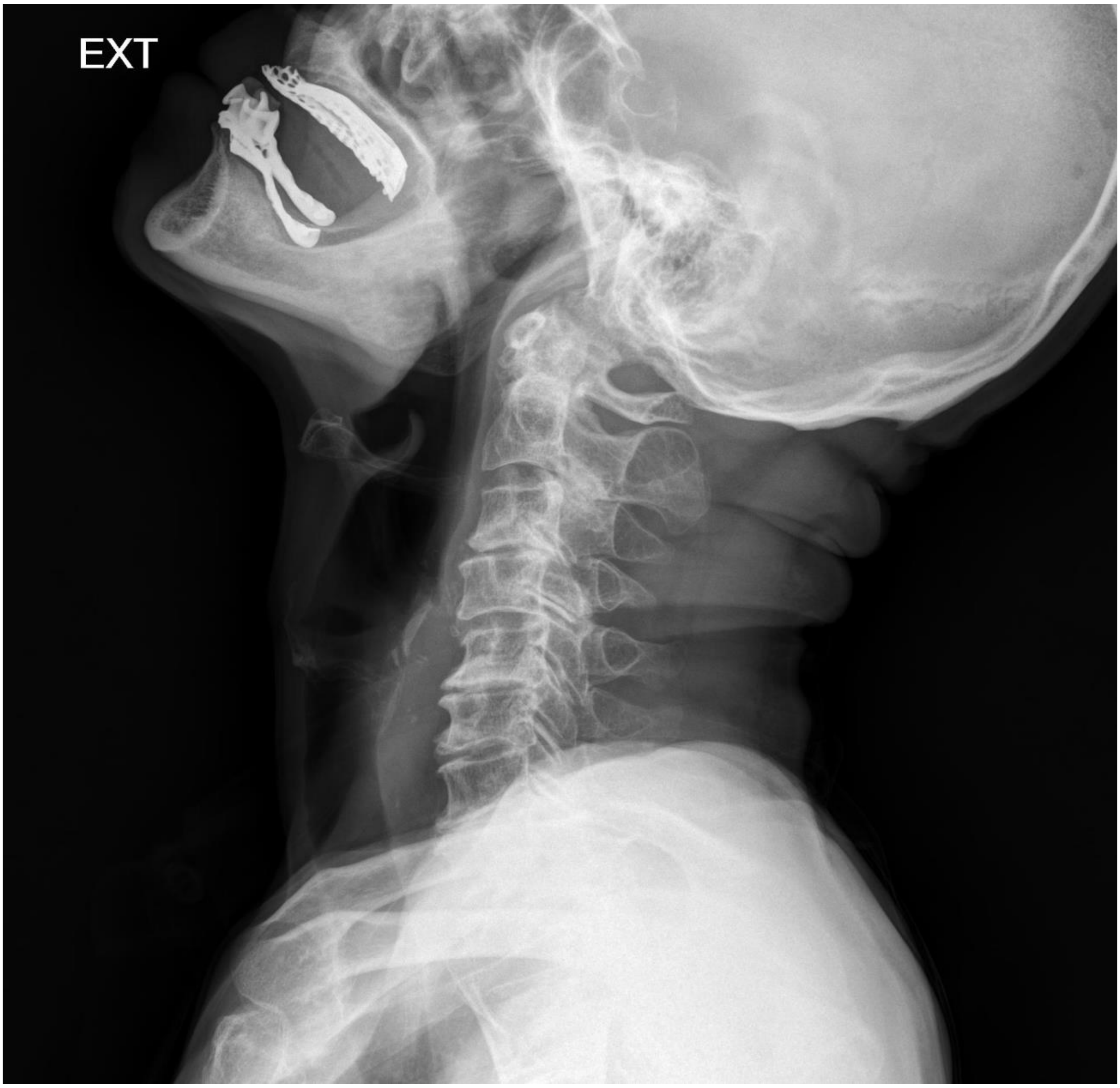

	C23	C34	C45	C56	C67	Total score
Endplate sclerosis	0	2	1	2	1	6
Disc space narrowing	0	3	1	3	2	9
Anterior osteophyte	0	1	1	2	1	5
Posterior osteophyte	0	1	1	1	1	4
Listhesis	1	1	1	1	1	5
Facet joint	1	0	1	1	1	4
Total score	2	8	6	10	7	33

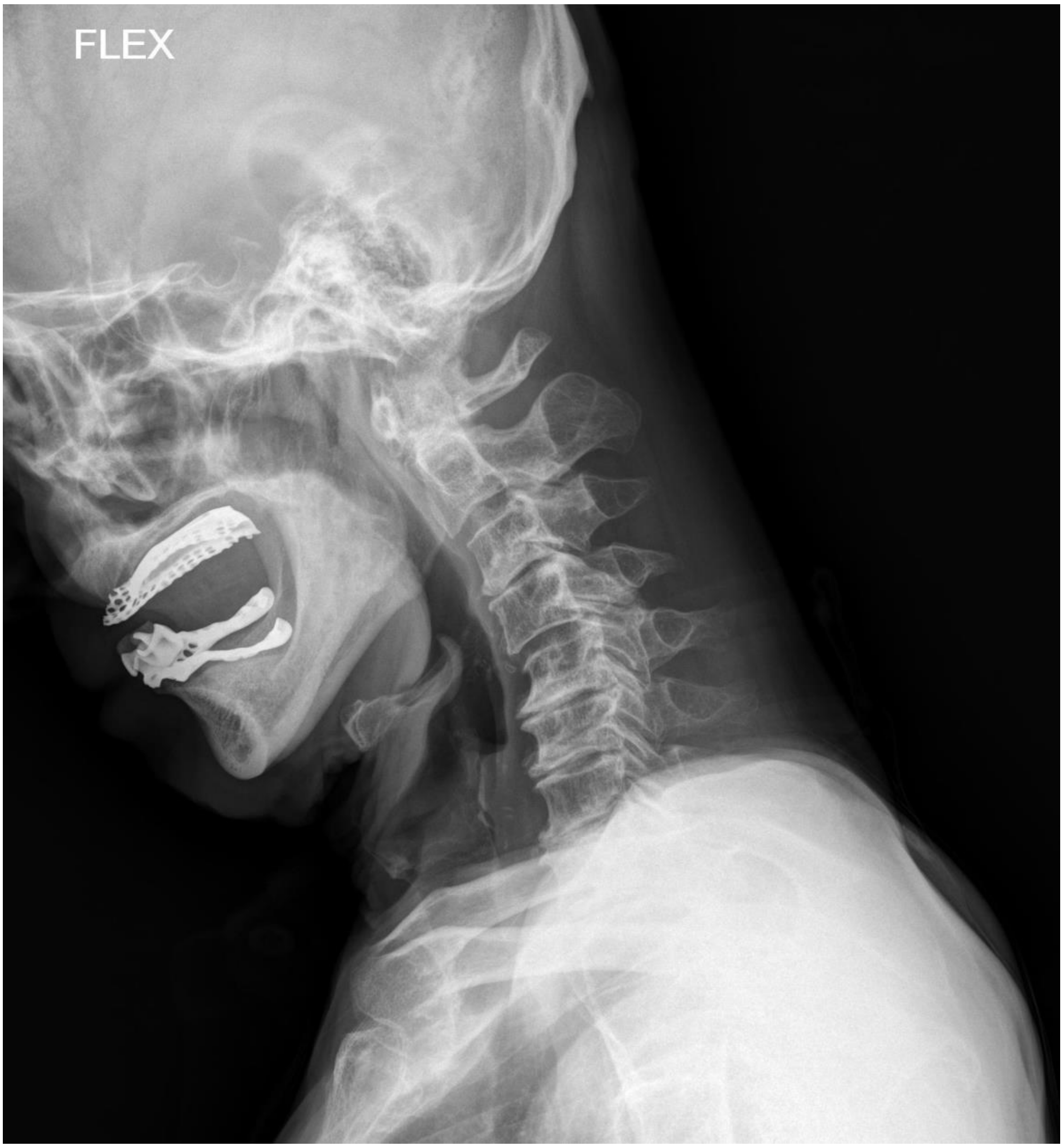

	C23	C34	C45	C56	C67	Total score
Endplate sclerosis	0	2	1	2	1	6
Disc space narrowing	0	3	1	3	2	9
Anterior osteophyte	0	1	1	2	1	5
Posterior osteophyte	0	1	1	1	1	4
Listhesis	1	1	1	1	1	5
Facet joint	1	0	1	1	1	4
Total score	2	8	6	10	7	33

Sample 2.

68/ male

C2-7 SVA: 11.9 mm

C2-7 ARA: 27.2°

	C23	C34	C45	C56	C67	Total score
Endplate sclerosis	0	1	1	2	2	6
Disc space narrowing	0	1	1	2	2	6
Anterior osteophyte	0	1	2	2	2	7
Posterior osteophyte	0	1	0	1	1	3
Listhesis	0	1	1	1	1	4
Facet joint	0	1	1	0	1	3
Total score	0	6	6	8	9	29

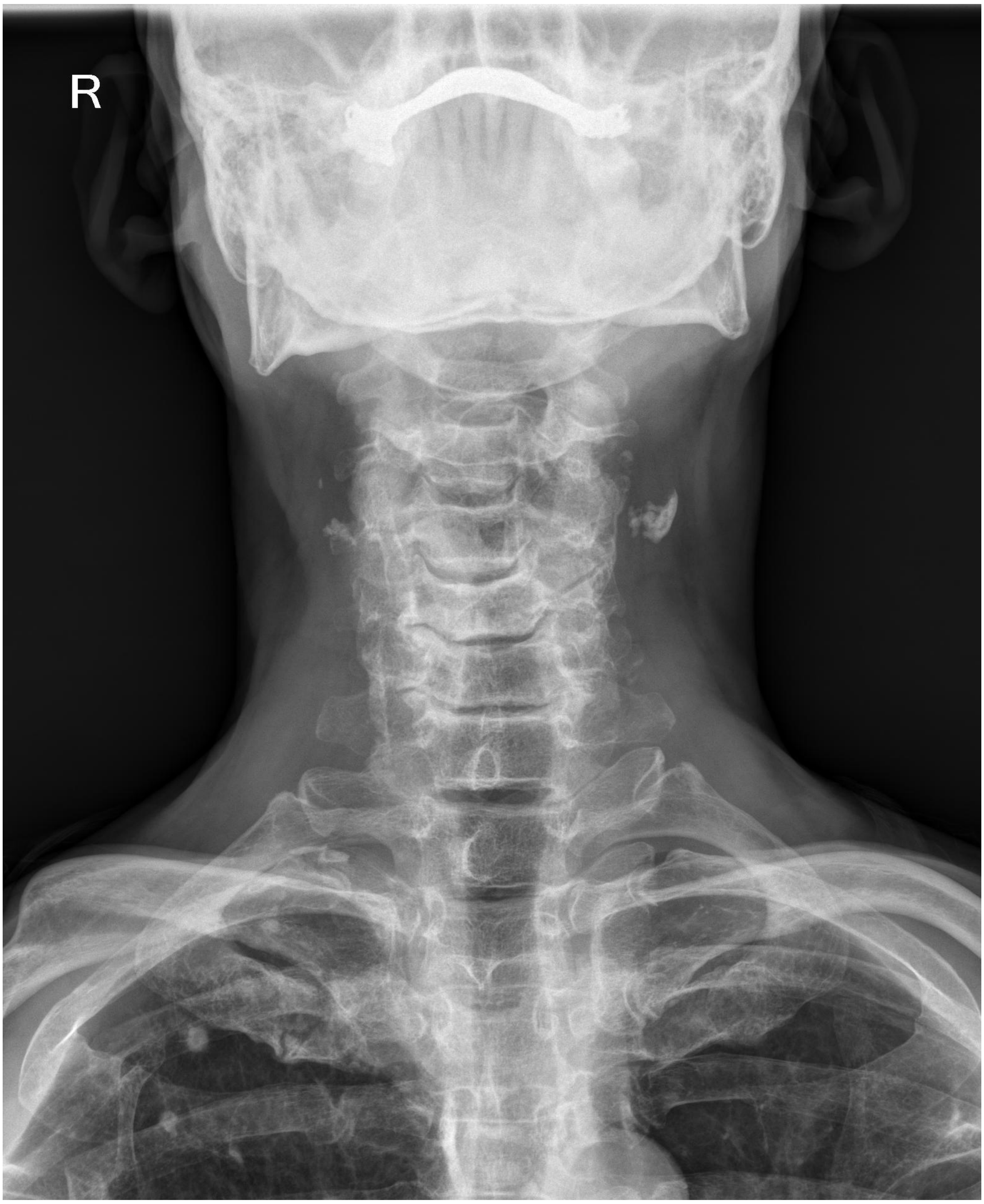

	C23	C34	C45	C56	C67	Total score
Endplate sclerosis	0	1	1	2	2	6
Disc space narrowing	0	1	1	2	2	6
Anterior osteophyte	0	1	2	2	2	7
Posterior osteophyte	0	1	0	1	1	3
Listhesis	0	1	1	1	1	4
Facet joint	0	1	1	0	1	3
Total score	0	6	6	8	9	29

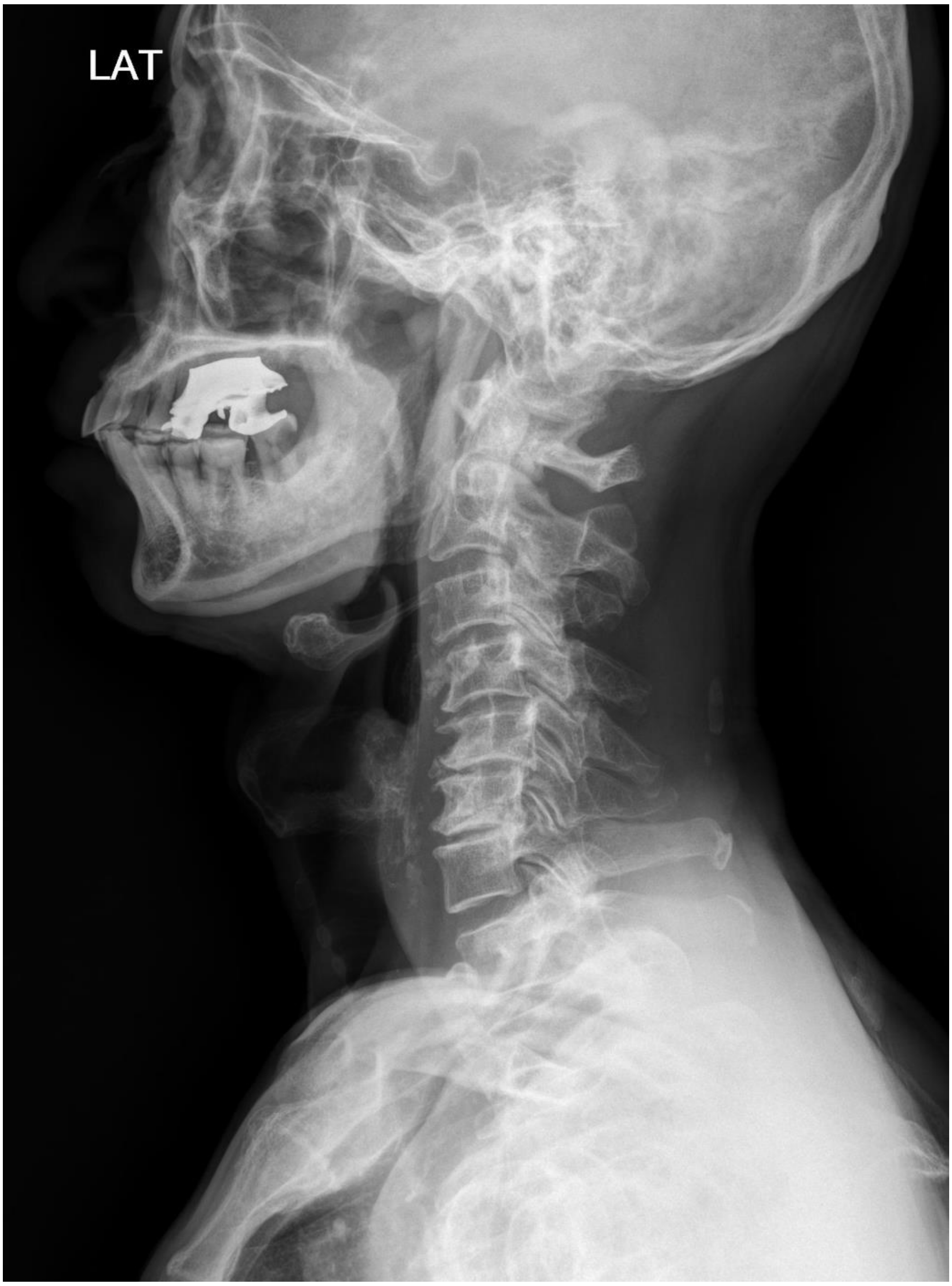

	C23	C34	C45	C56	C67	Total score
Endplate sclerosis	0	1	1	2	2	6
Disc space narrowing	0	1	1	2	2	6
Anterior osteophyte	0	1	2	2	2	7
Posterior osteophyte	0	1	0	1	1	3
Listhesis	0	1	1	1	1	4
Facet joint	0	1	1	0	1	3
Total score	0	6	6	8	9	29

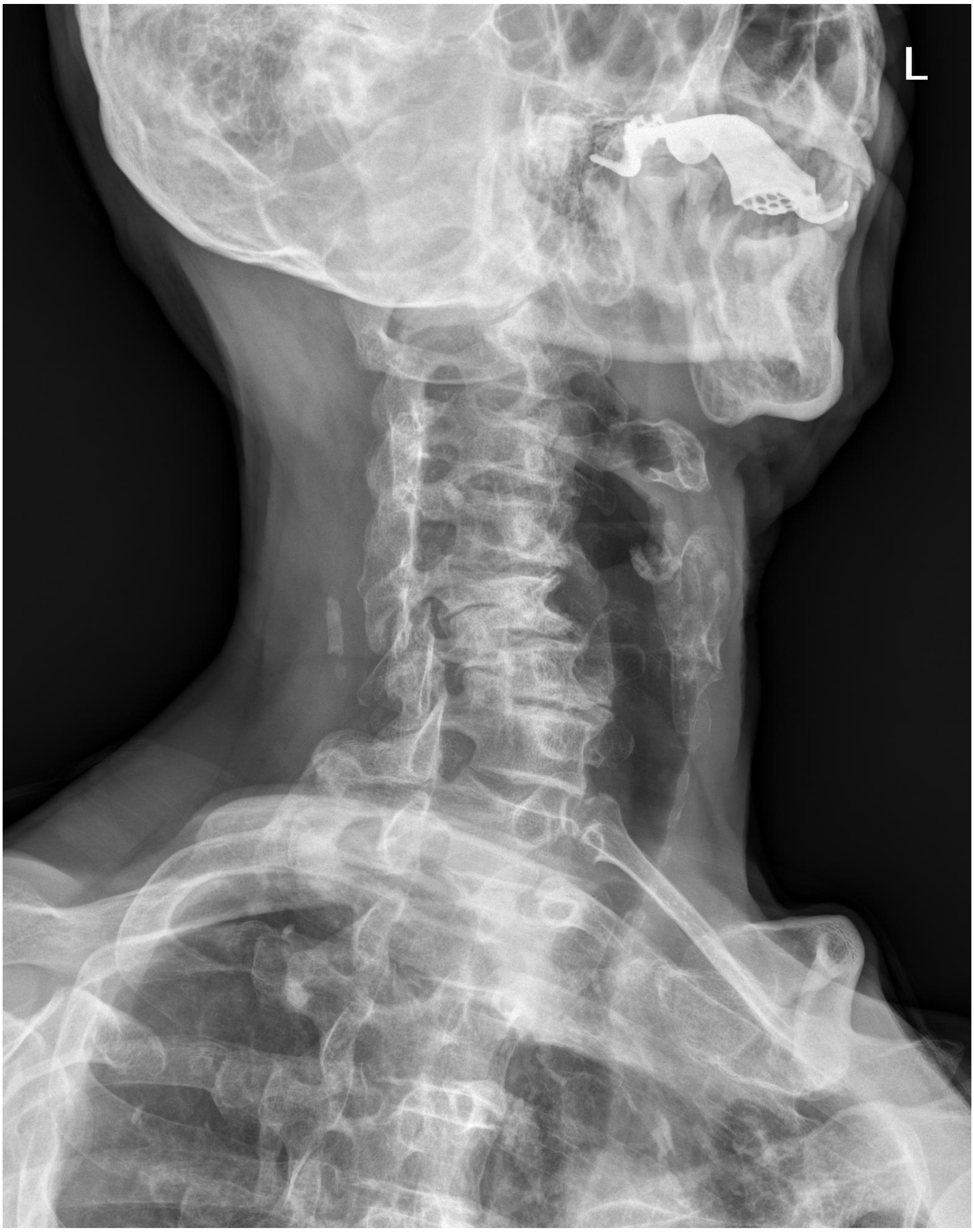

	C23	C34	C45	C56	C67	Total score
Endplate sclerosis	0	1	1	2	2	6
Disc space narrowing	0	1	1	2	2	6
Anterior osteophyte	0	1	2	2	2	7
Posterior osteophyte	0	1	0	1	1	3
Listhesis	0	1	1	1	1	4
Facet joint	0	1	1	0	1	3
Total score	0	6	6	8	9	29

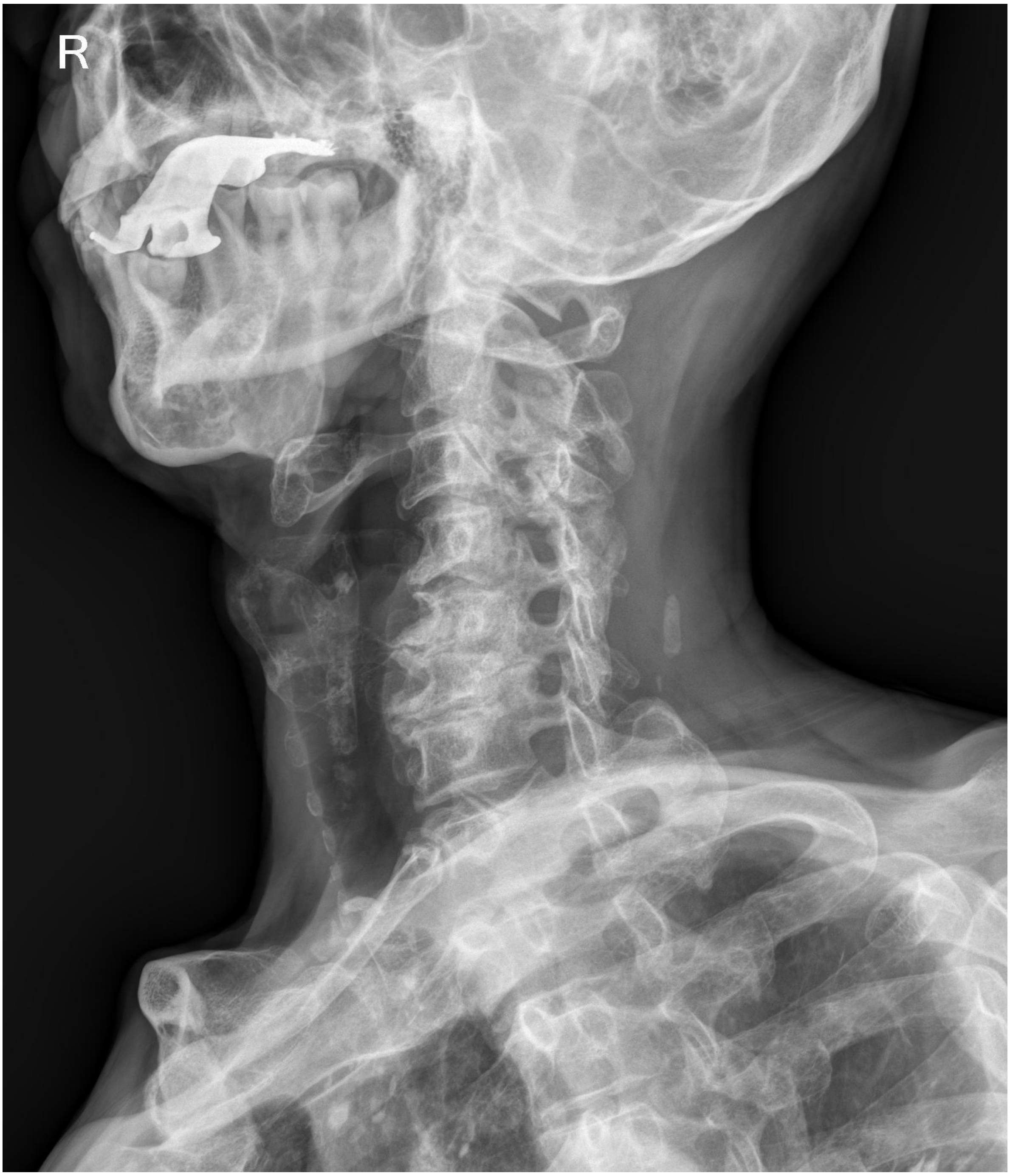

	C23	C34	C45	C56	C67	Total score
Endplate sclerosis	0	1	1	2	2	6
Disc space narrowing	0	1	1	2	2	6
Anterior osteophyte	0	1	2	2	2	7
Posterior osteophyte	0	1	0	1	1	3
Listhesis	0	1	1	1	1	4
Facet joint	0	1	1	0	1	3
Total score	0	6	6	8	9	29

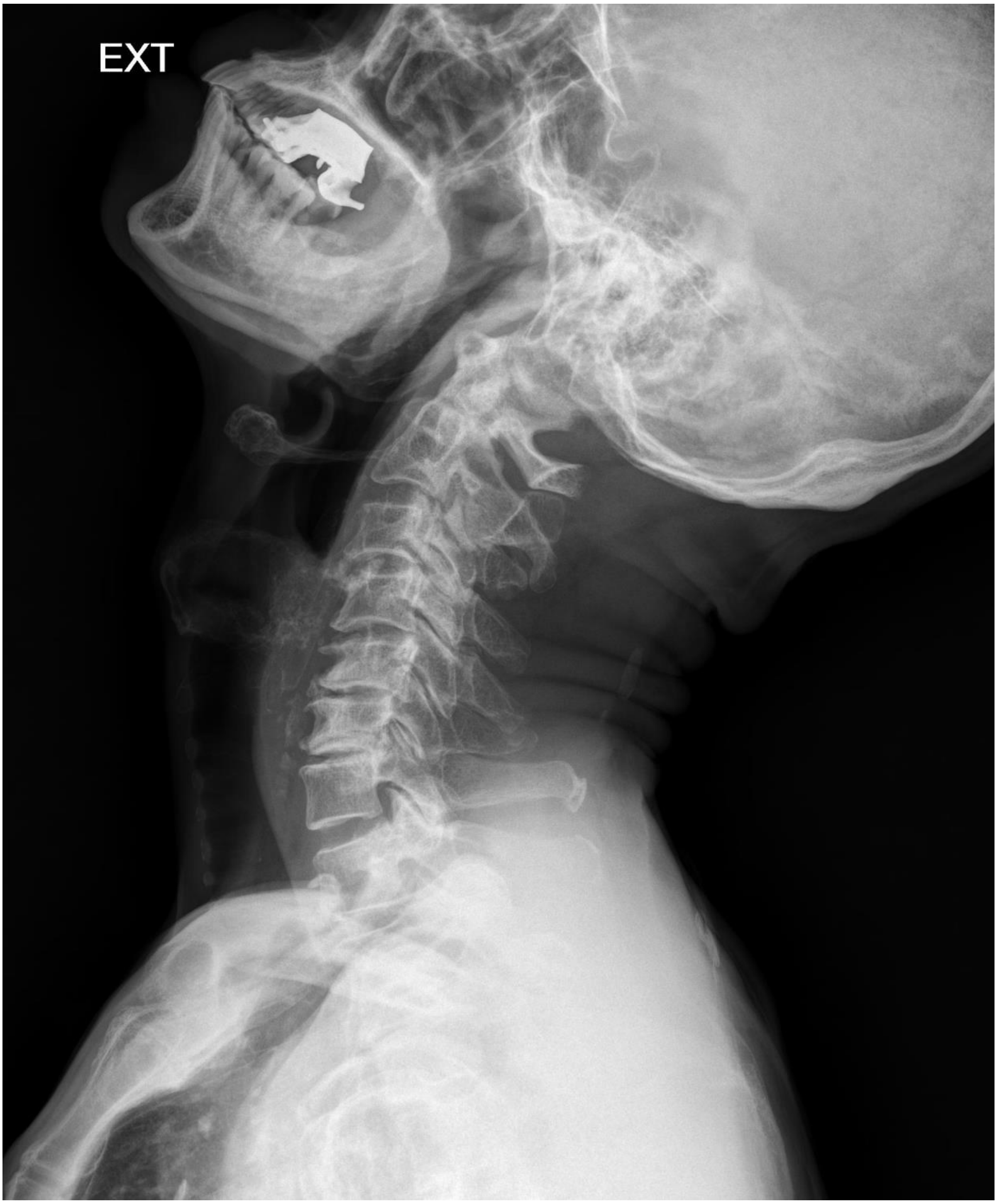

	C23	C34	C45	C56	C67	Total score
Endplate sclerosis	0	1	1	2	2	6
Disc space narrowing	0	1	1	2	2	6
Anterior osteophyte	0	1	2	2	2	7
Posterior osteophyte	0	1	0	1	1	3
Listhesis	0	1	1	1	1	4
Facet joint	0	1	1	0	1	3
Total score	0	6	6	8	9	29

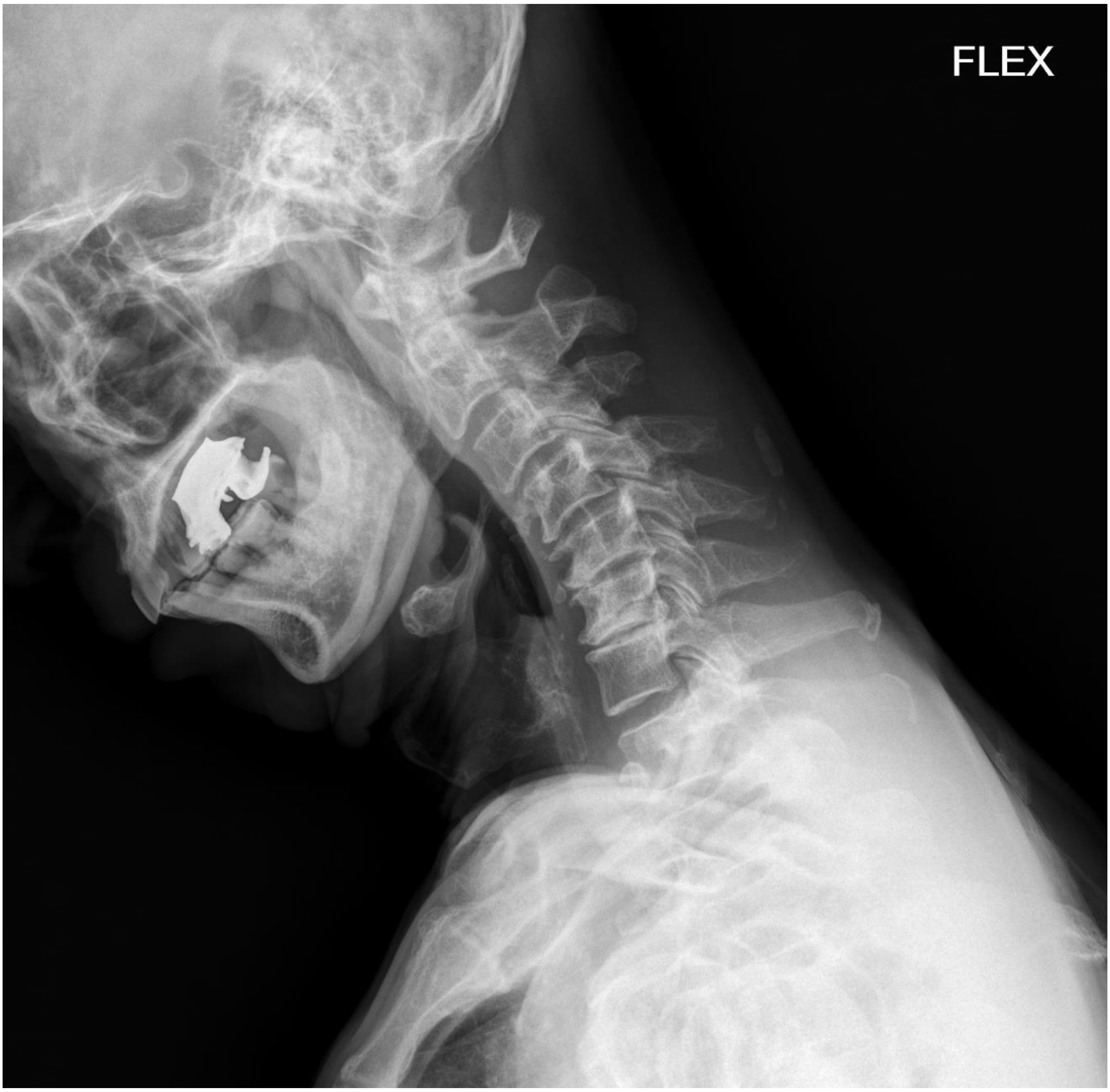

	C23	C34	C45	C56	C67	Total score
Endplate sclerosis	0	1	1	2	2	6
Disc space narrowing	0	1	1	2	2	6
Anterior osteophyte	0	1	2	2	2	7
Posterior osteophyte	0	1	0	1	1	3
Listhesis	0	1	1	1	1	4
Facet joint	0	1	1	0	1	3
Total score	0	6	6	8	9	29

Sample 3.

68/ male

C2-7 SVA: 28.8 mm

C2-7 ARA: -7.2°

	C23	C34	C45	C56	C67	Total score
Endplate sclerosis	0	1	1	1	1	4
Disc space narrowing	0	1	2	2	1	6
Anterior osteophyte	0	1	1	2	1	5
Posterior osteophyte	0	1	1	1	1	4
Listhesis	0	1	1	1	1	4
Facet joint	0	1	0	0	0	1
Total score	0	6	6	7	5	24

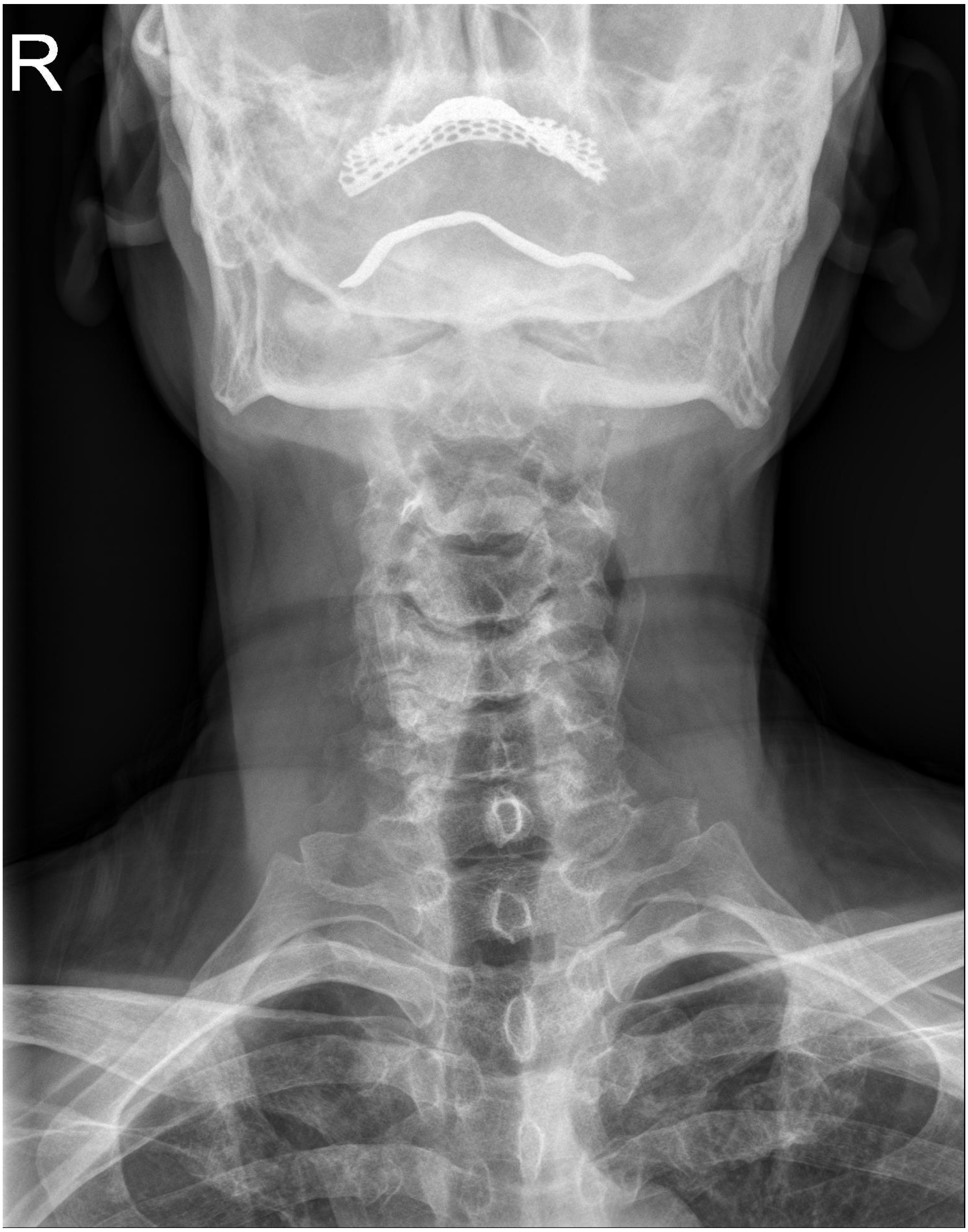

	C23	C34	C45	C56	C67	Total score
Endplate sclerosis	0	1	1	1	1	4
Disc space narrowing	0	1	2	2	1	6
Anterior osteophyte	0	1	1	2	1	5
Posterior osteophyte	0	1	1	1	1	4
Listhesis	0	1	1	1	1	4
Facet joint	0	1	0	0	0	1
Total score	0	6	6	7	5	24

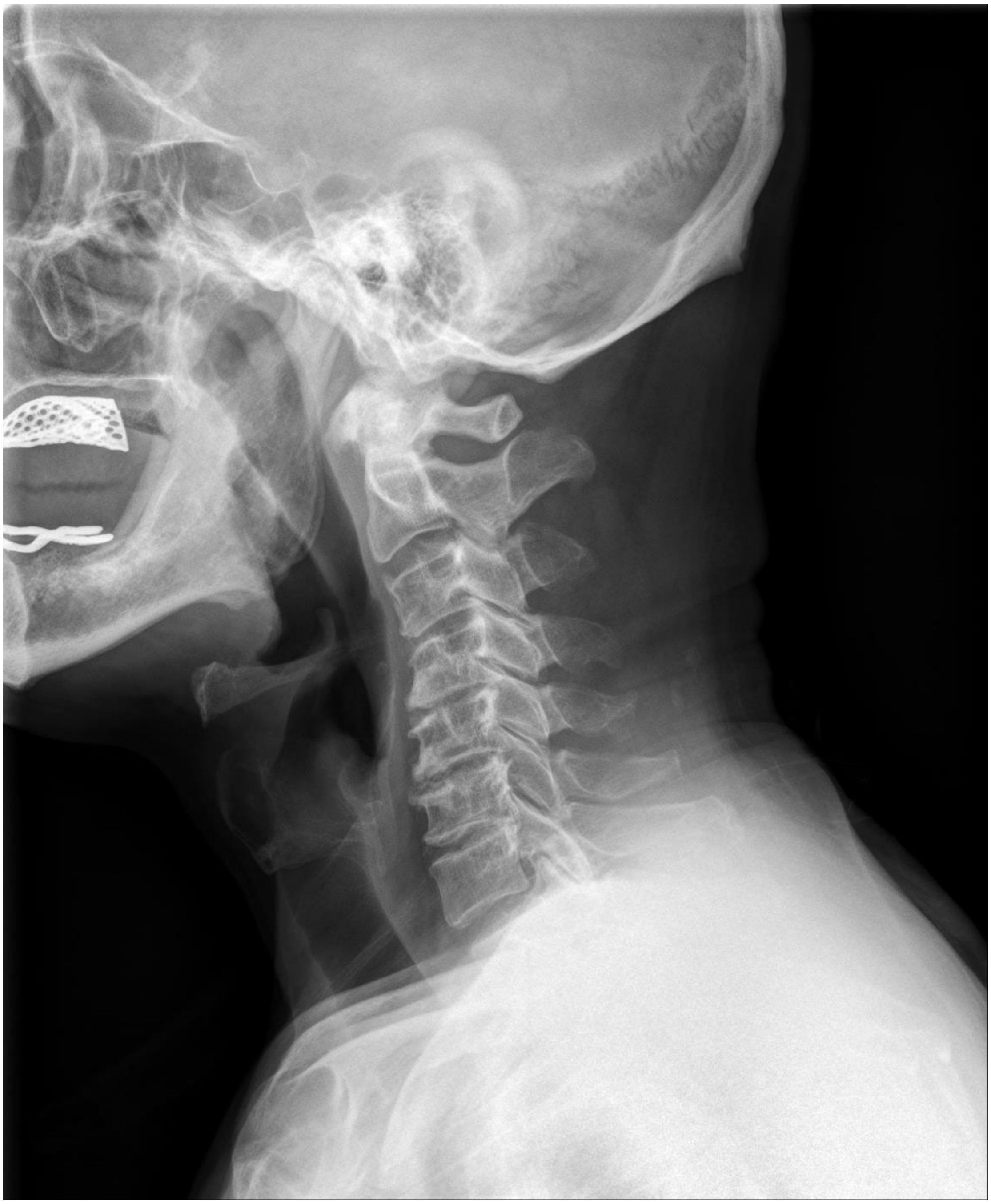

	C23	C34	C45	C56	C67	Total score
Endplate sclerosis	0	1	1	1	1	4
Disc space narrowing	0	1	2	2	1	6
Anterior osteophyte	0	1	1	2	1	5
Posterior osteophyte	0	1	1	1	1	4
Listhesis	0	1	1	1	1	4
Facet joint	0	1	0	0	0	1
Total score	0	6	6	7	5	24

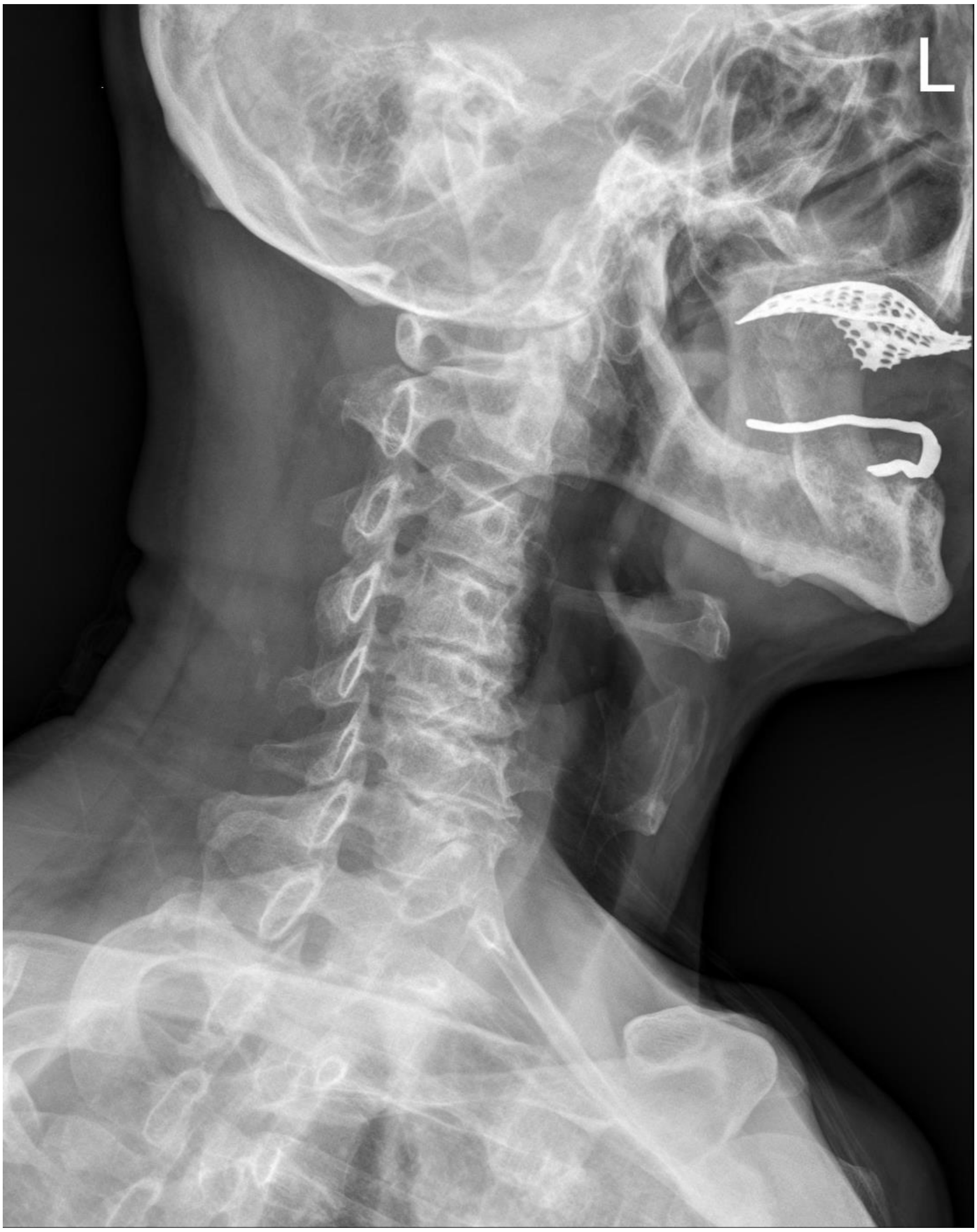

	C23	C34	C45	C56	C67	Total score
Endplate sclerosis	0	1	1	1	1	4
Disc space narrowing	0	1	2	2	1	6
Anterior osteophyte	0	1	1	2	1	5
Posterior osteophyte	0	1	1	1	1	4
Listhesis	0	1	1	1	1	4
Facet joint	0	1	0	0	0	1
Total score	0	6	6	7	5	24

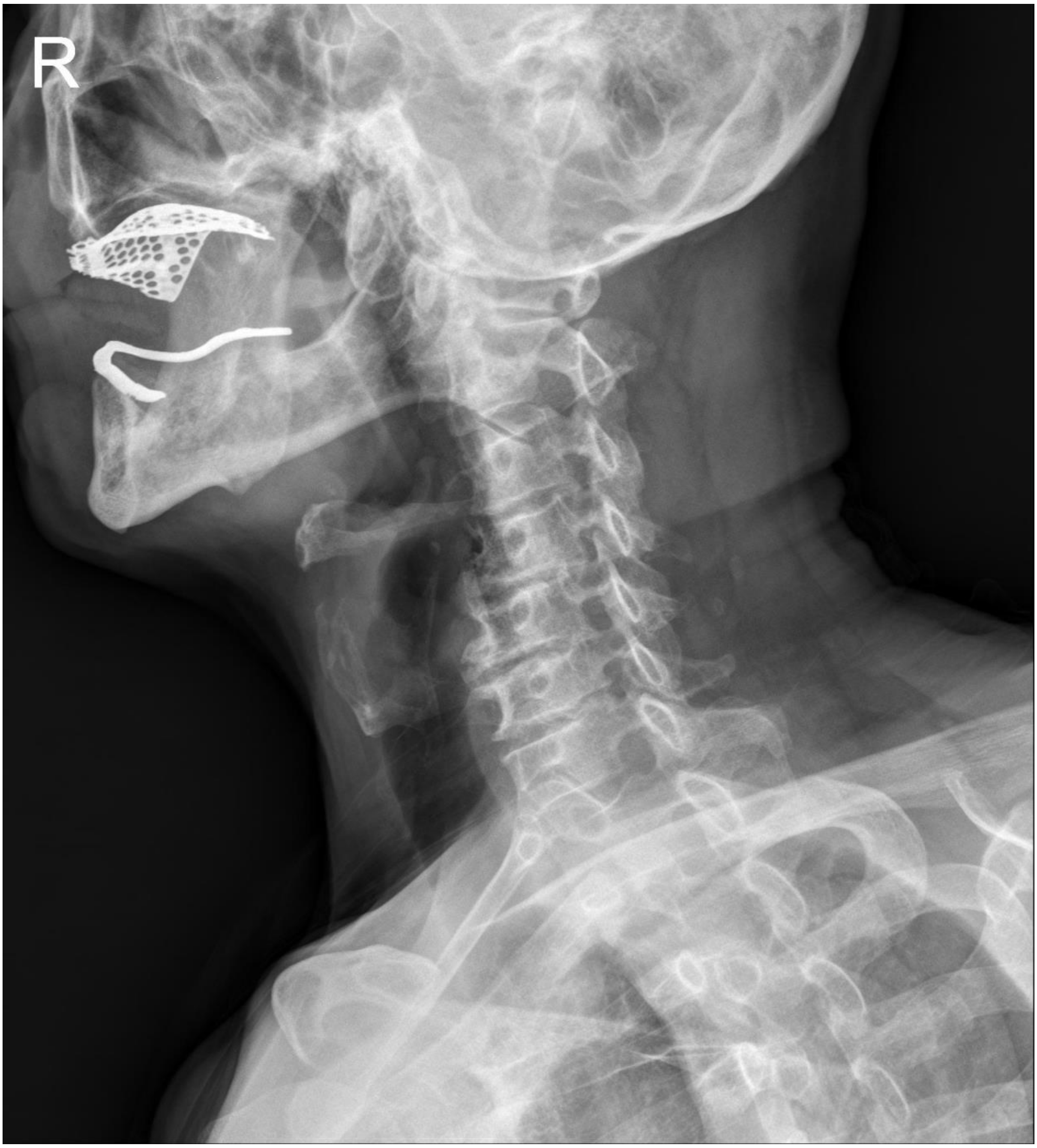

	C23	C34	C45	C56	C67	Total score
Endplate sclerosis	0	1	1	1	1	4
Disc space narrowing	0	1	2	2	1	6
Anterior osteophyte	0	1	1	2	1	5
Posterior osteophyte	0	1	1	1	1	4
Listhesis	0	1	1	1	1	4
Facet joint	0	1	0	0	0	1
Total score	0	6	6	7	5	24

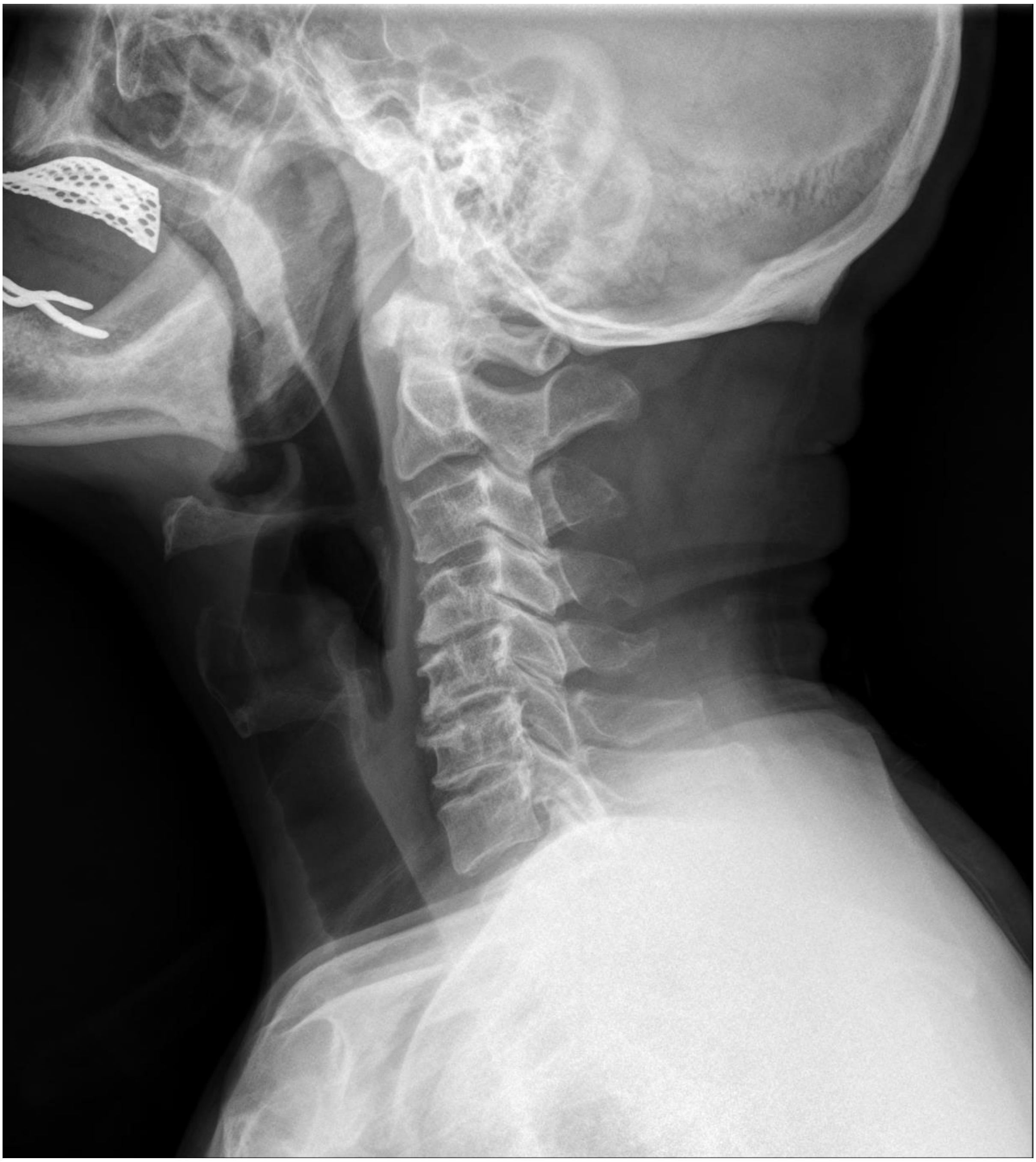

	C23	C34	C45	C56	C67	Total score
Endplate sclerosis	0	1	1	1	1	4
Disc space narrowing	0	1	2	2	1	6
Anterior osteophyte	0	1	1	2	1	5
Posterior osteophyte	0	1	1	1	1	4
Listhesis	0	1	1	1	1	4
Facet joint	0	1	0	0	0	1
Total score	0	6	6	7	5	24

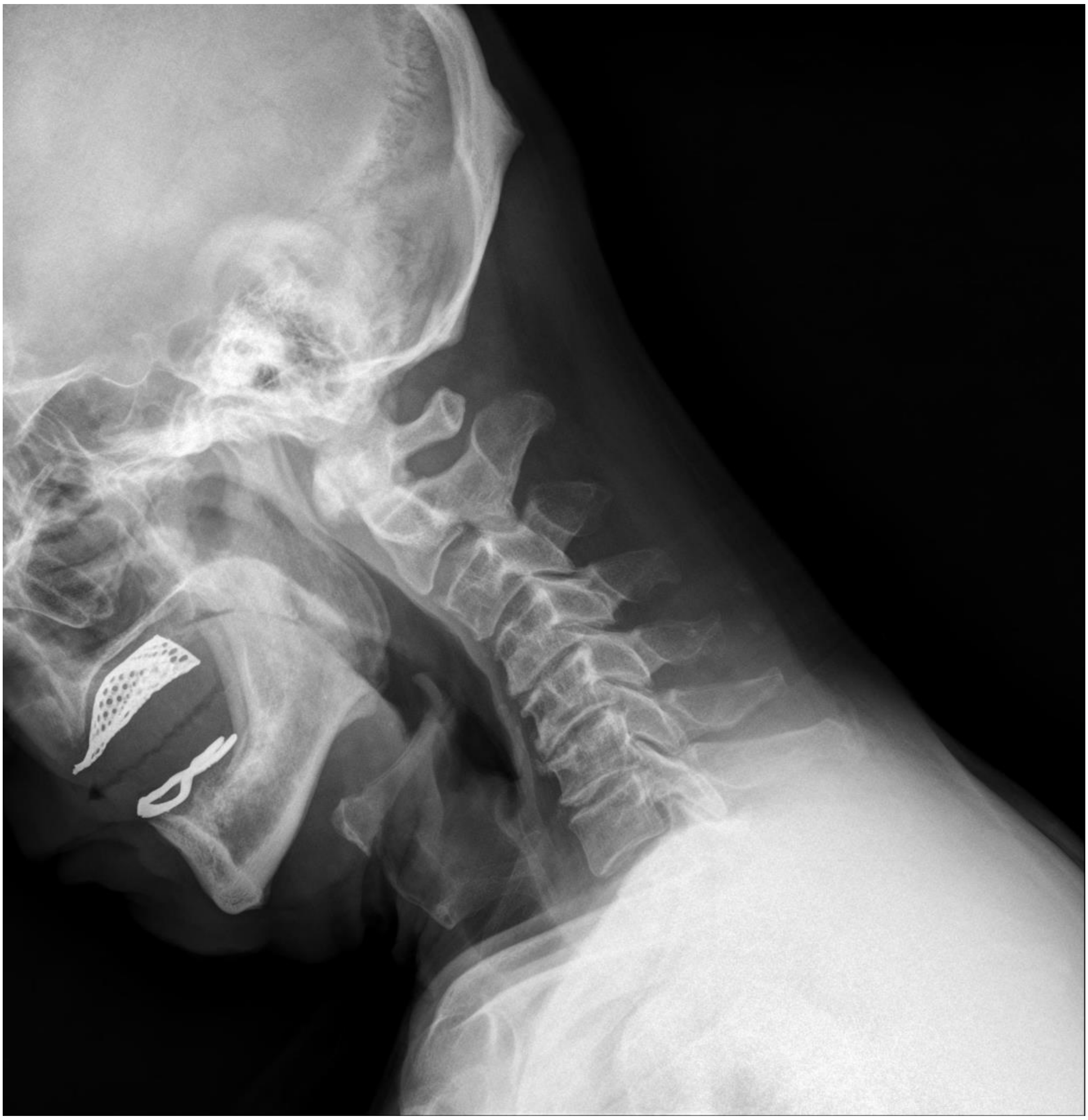

	C23	C34	C45	C56	C67	Total score
Endplate sclerosis	0	1	1	1	1	4
Disc space narrowing	0	1	2	2	1	6
Anterior osteophyte	0	1	1	2	1	5
Posterior osteophyte	0	1	1	1	1	4
Listhesis	0	1	1	1	1	4
Facet joint	0	1	0	0	0	1
Total score	0	6	6	7	5	24

Sample 4.

68/ male

C2-7 SVA: 34.4mm

C2-7 ARA: 22.2°

	C23	C34	C45	C56	C67	Total score
Endplate sclerosis	0	1	1	1	0	3
Disc space narrowing	0	0	1	2	0	3
Anterior osteophyte	0	1	2	2	0	5
Posterior osteophyte	0	0	1	1	0	2
Listhesis	0	0	0	1	1	2
Facet joint	1	2	1	0	1	5
Total score	1	4	6	7	2	20

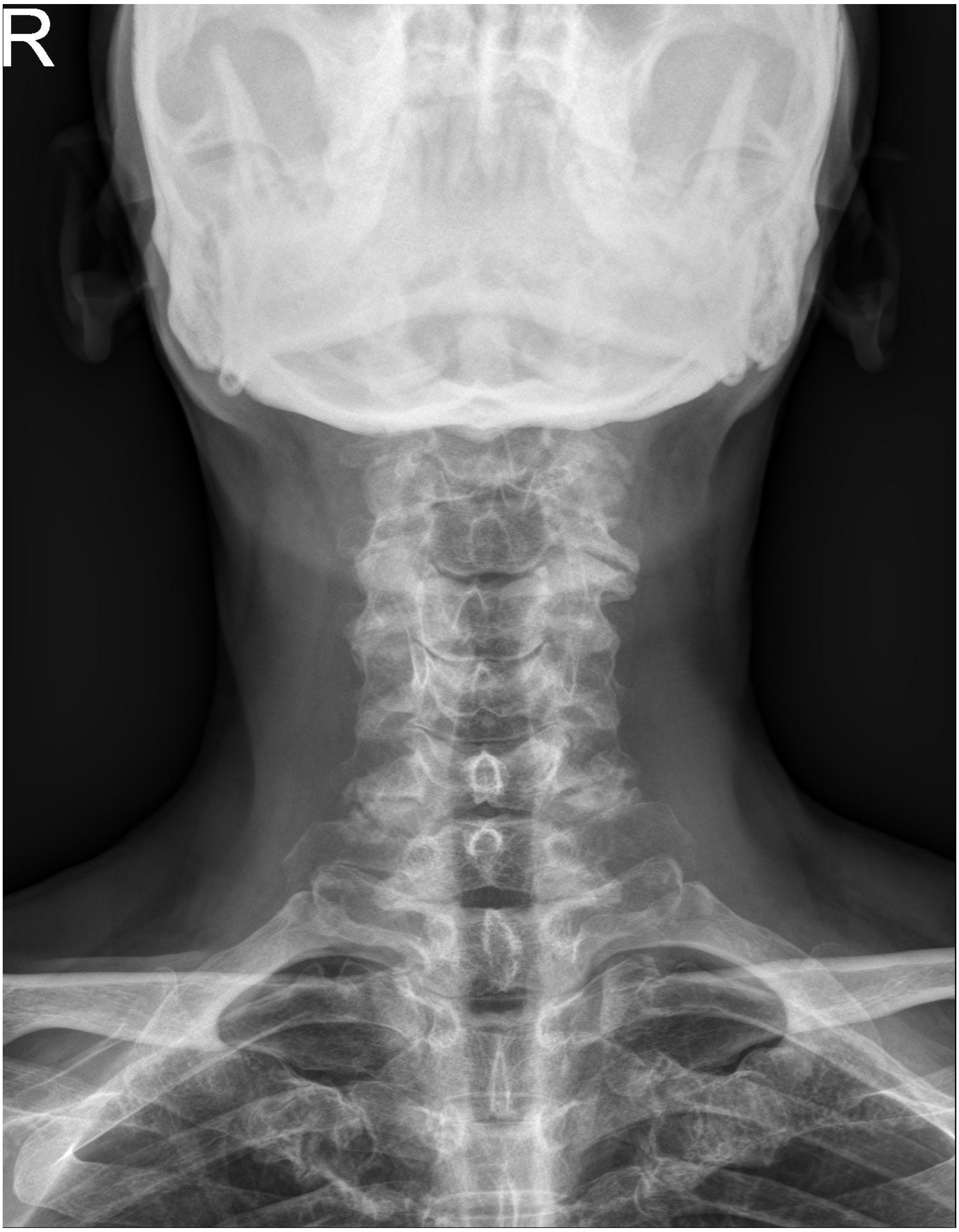

	C23	C34	C45	C56	C67	Total score
Endplate sclerosis	0	1	1	1	0	3
Disc space narrowing	0	0	1	2	0	3
Anterior osteophyte	0	1	2	2	0	5
Posterior osteophyte	0	0	1	1	0	2
Listhesis	0	0	0	1	1	2
Facet joint	1	2	1	0	1	5
Total score	1	4	6	7	2	20

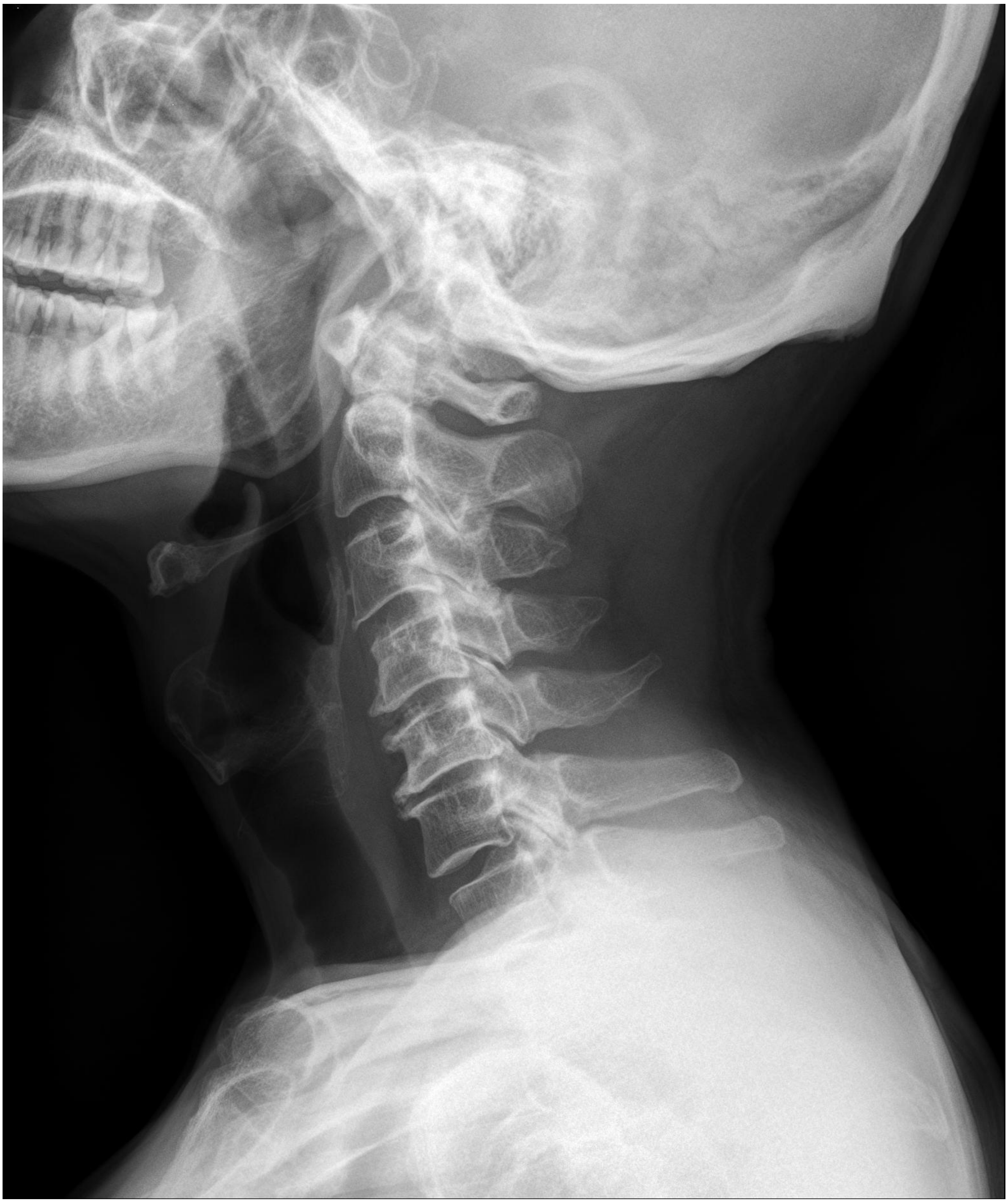

	C23	C34	C45	C56	C67	Total score
Endplate sclerosis	0	1	1	1	0	3
Disc space narrowing	0	0	1	2	0	3
Anterior osteophyte	0	1	2	2	0	5
Posterior osteophyte	0	0	1	1	0	2
Listhesis	0	0	0	1	1	2
Facet joint	1	2	1	0	1	5
Total score	1	4	6	7	2	20

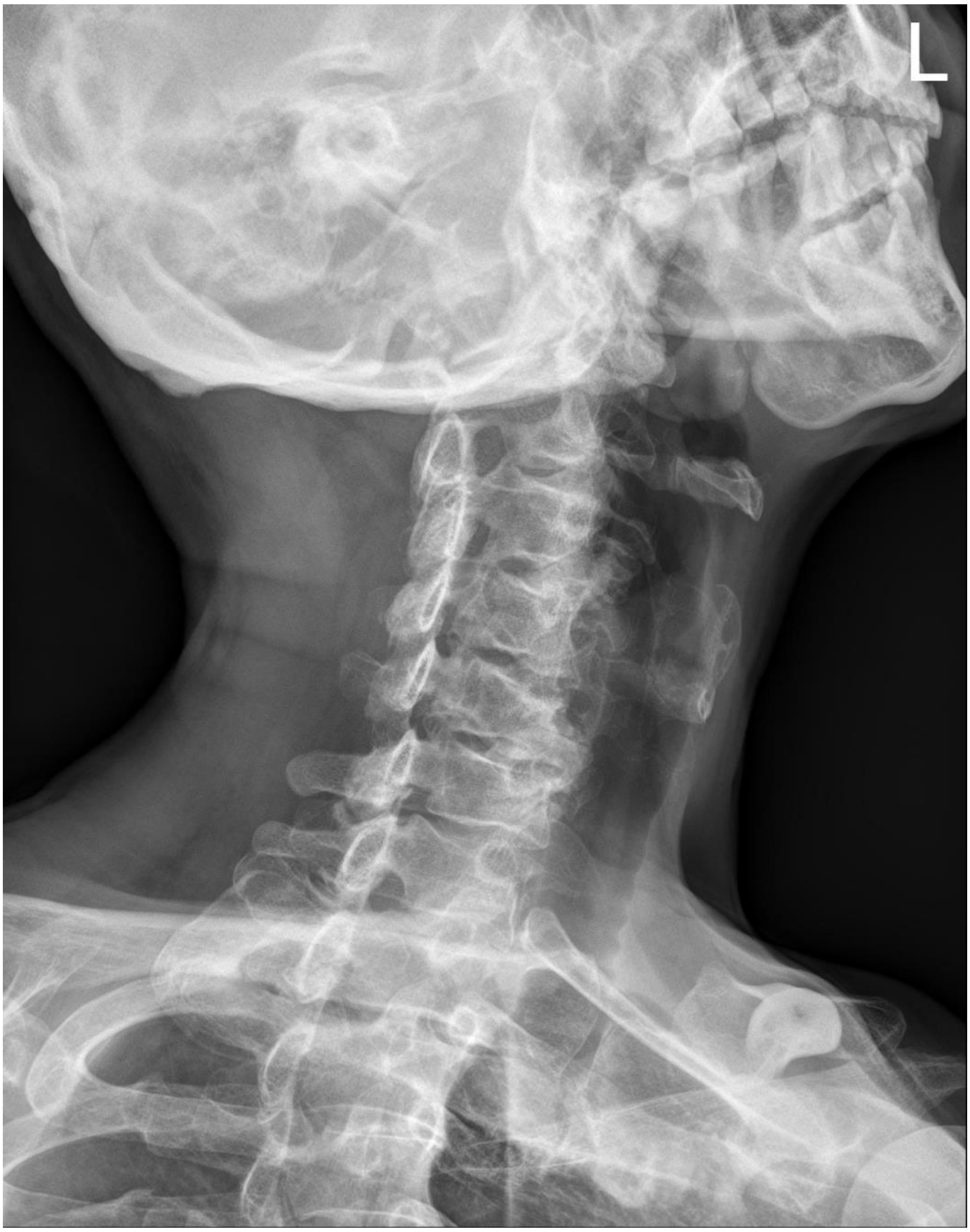

	C23	C34	C45	C56	C67	Total score
Endplate sclerosis	0	1	1	1	0	3
Disc space narrowing	0	0	1	2	0	3
Anterior osteophyte	0	1	2	2	0	5
Posterior osteophyte	0	0	1	1	0	2
Listhesis	0	0	0	1	1	2
Facet joint	1	2	1	0	1	5
Total score	1	4	6	7	2	20

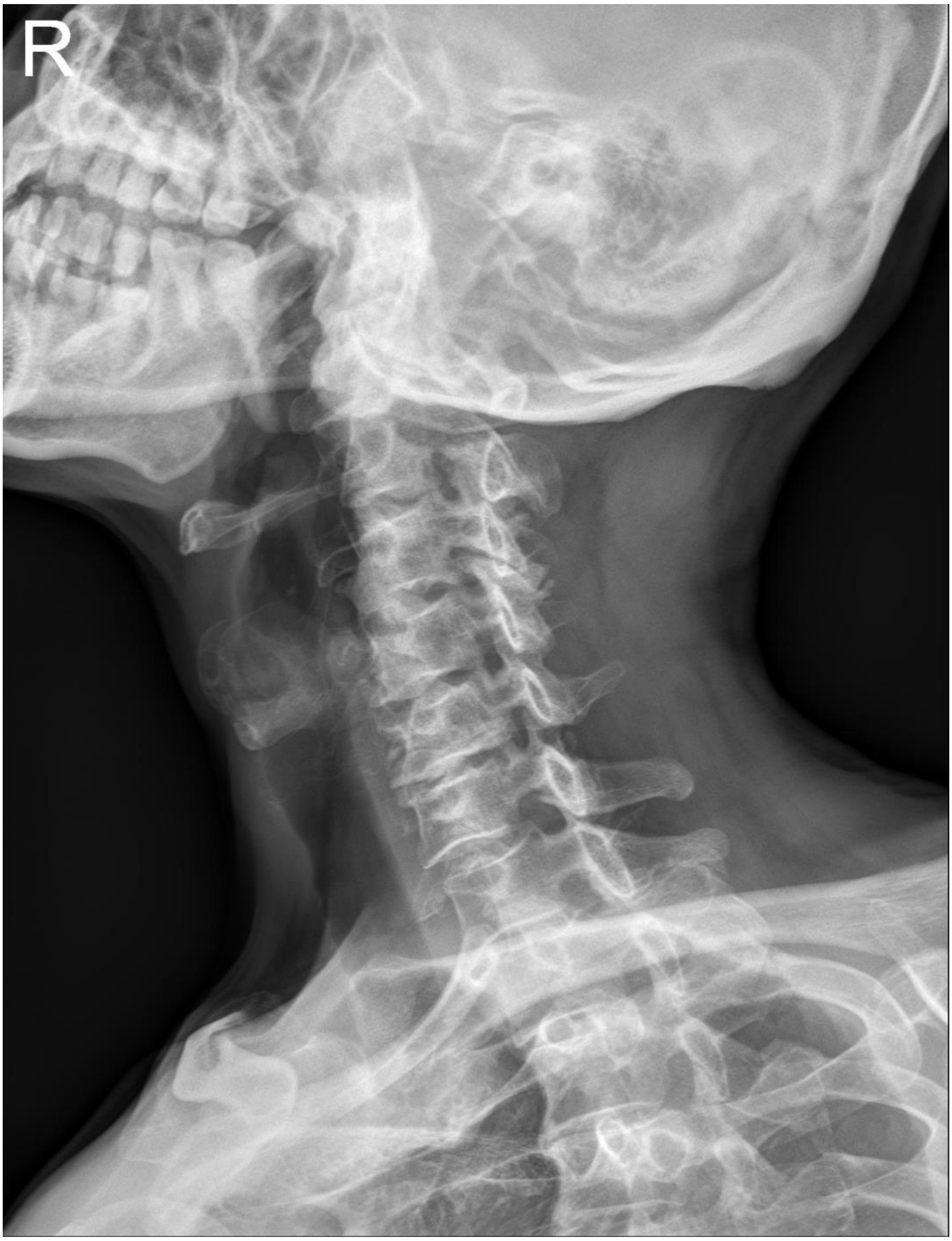

	C23	C34	C45	C56	C67	Total score
Endplate sclerosis	0	1	1	1	0	3
Disc space narrowing	0	0	1	2	0	3
Anterior osteophyte	0	1	2	2	0	5
Posterior osteophyte	0	0	1	1	0	2
Listhesis	0	0	0	1	1	2
Facet joint	1	2	1	0	1	5
Total score	1	4	6	7	2	20

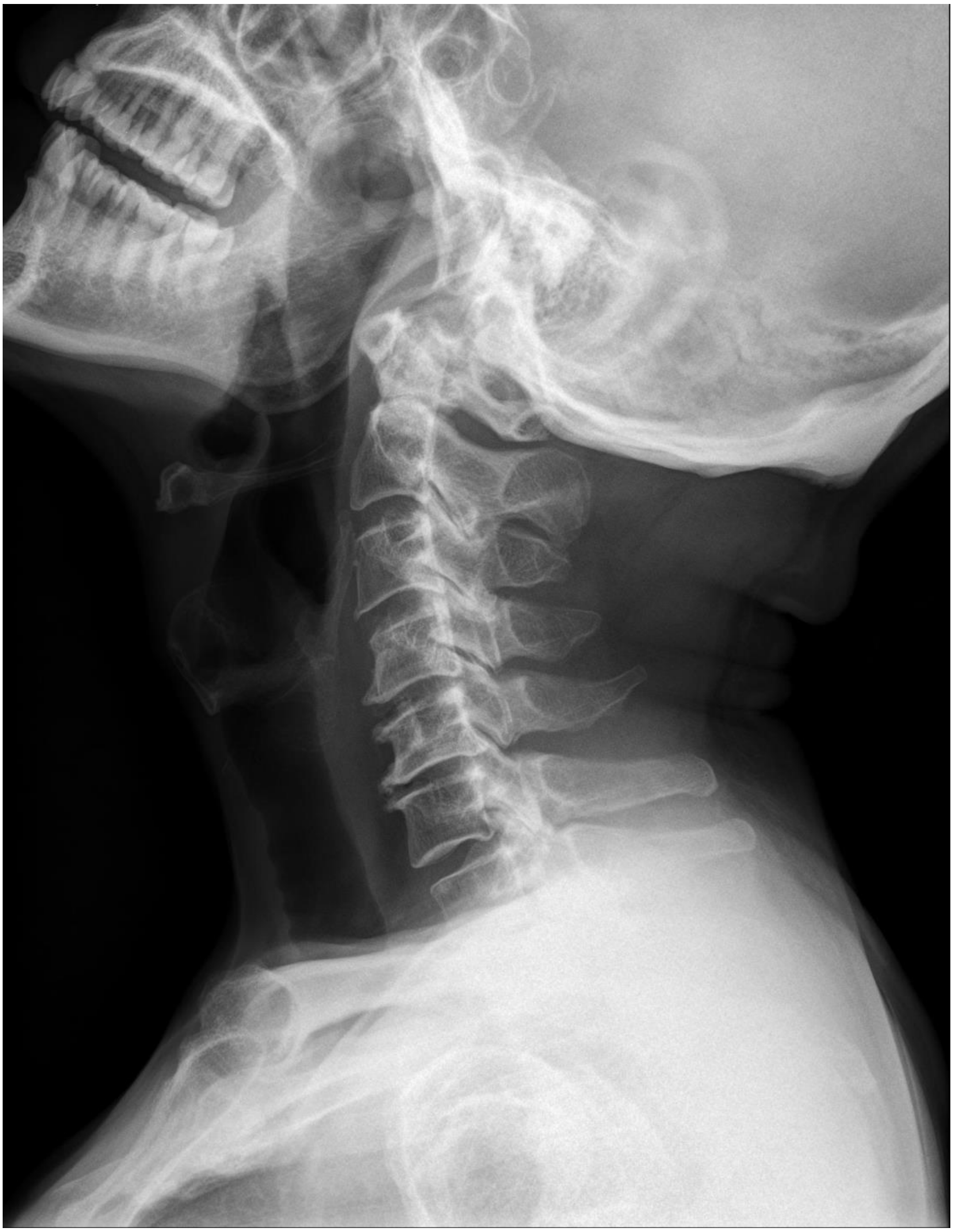

	C23	C34	C45	C56	C67	Total score
Endplate sclerosis	0	1	1	1	0	3
Disc space narrowing	0	0	1	2	0	3
Anterior osteophyte	0	1	2	2	0	5
Posterior osteophyte	0	0	1	1	0	2
Listhesis	0	0	0	1	1	2
Facet joint	1	2	1	0	1	5
Total score	1	4	6	7	2	20

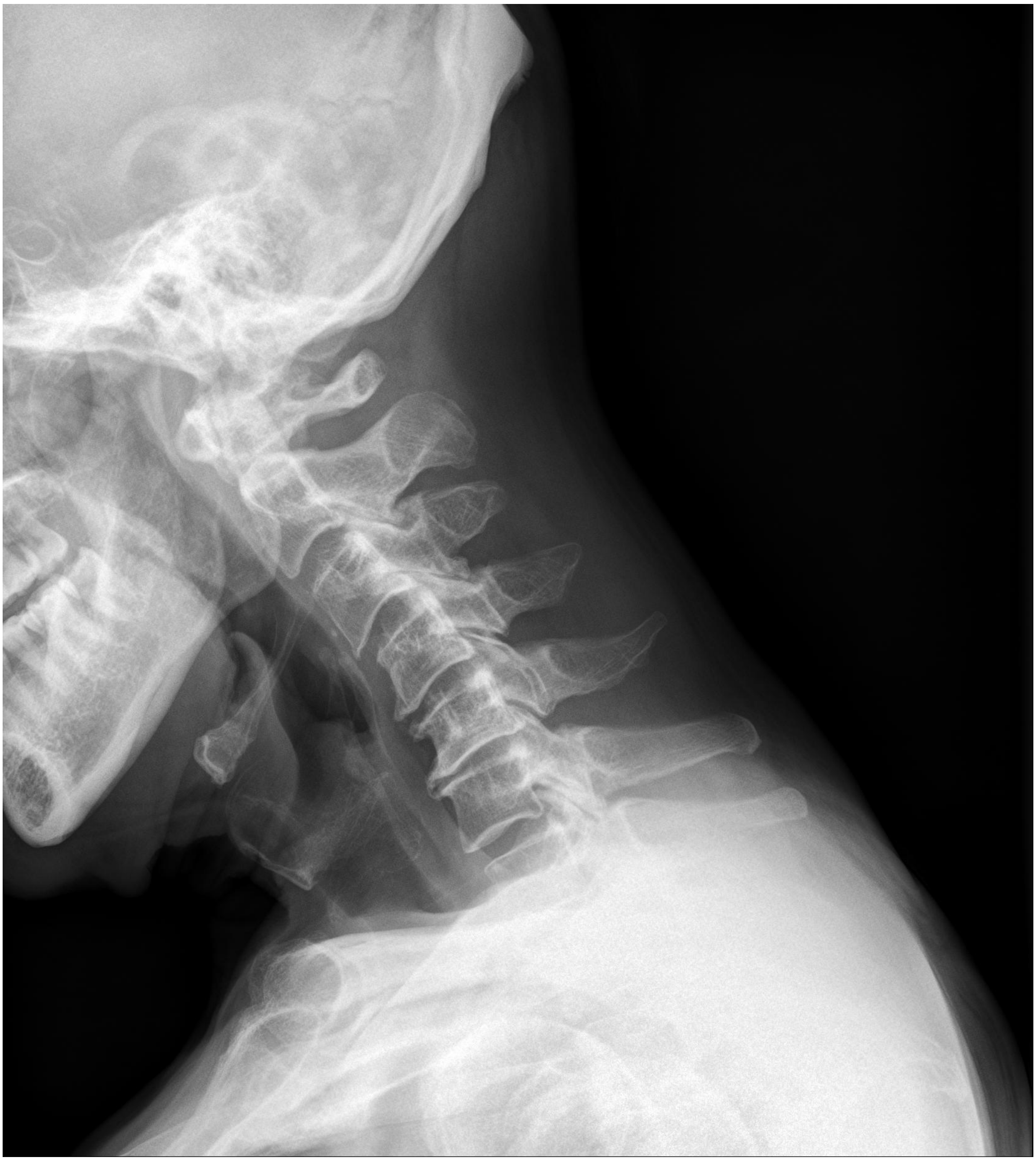

	C23	C34	C45	C56	C67	Total score
Endplate sclerosis	0	1	1	1	0	3
Disc space narrowing	0	0	1	2	0	3
Anterior osteophyte	0	1	2	2	0	5
Posterior osteophyte	0	0	1	1	0	2
Listhesis	0	0	0	1	1	2
Facet joint	1	2	1	0	1	5
Total score	1	4	6	7	2	20

Sample 5.

75/ male

C2-7 SVA: 26.0 mm

C2-7 ARA: 30.3°

	C23	C34	C45	C56	C67	Total score
Endplate sclerosis	0	1	1	1	0	3
Disc space narrowing	0	1	1	2	0	4
Anterior osteophyte	0	1	1	2	1	5
Posterior osteophyte	0	1	1	1	0	3
Listhesis	0	0	0	0	0	0
Facet joint	0	0	0	0	0	0
Total score	0	4	4	6	1	15

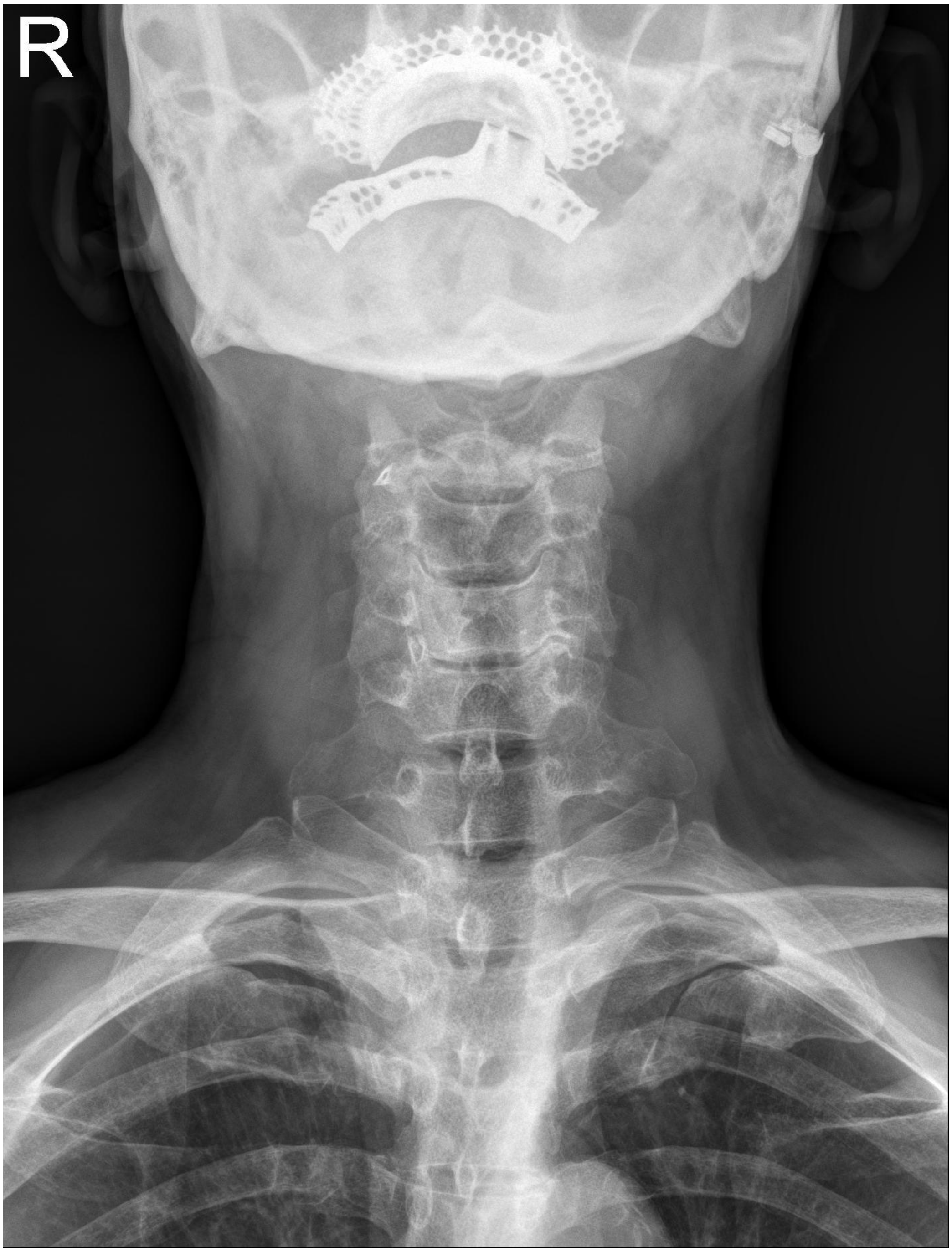

	C23	C34	C45	C56	C67	Total score
Endplate sclerosis	0	1	1	1	0	3
Disc space narrowing	0	1	1	2	0	4
Anterior osteophyte	0	1	1	2	1	5
Posterior osteophyte	0	1	1	1	0	3
Listhesis	0	0	0	0	0	0
Facet joint	0	0	0	0	0	0
Total score	0	4	4	6	1	15

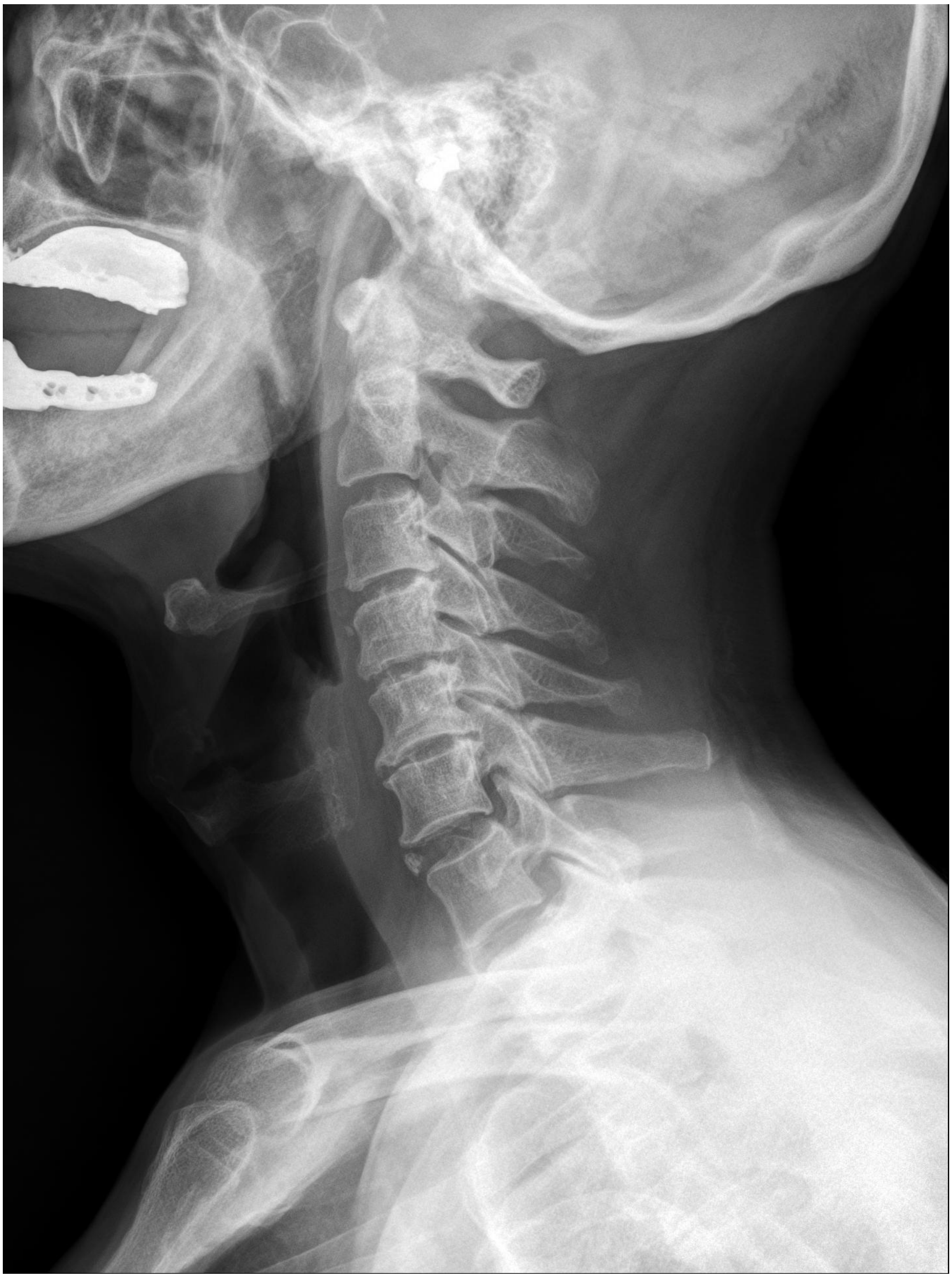

	C23	C34	C45	C56	C67	Total score
Endplate sclerosis	0	1	1	1	0	3
Disc space narrowing	0	1	1	2	0	4
Anterior osteophyte	0	1	1	2	1	5
Posterior osteophyte	0	1	1	1	0	3
Listhesis	0	0	0	0	0	0
Facet joint	0	0	0	0	0	0
Total score	0	4	4	6	1	15

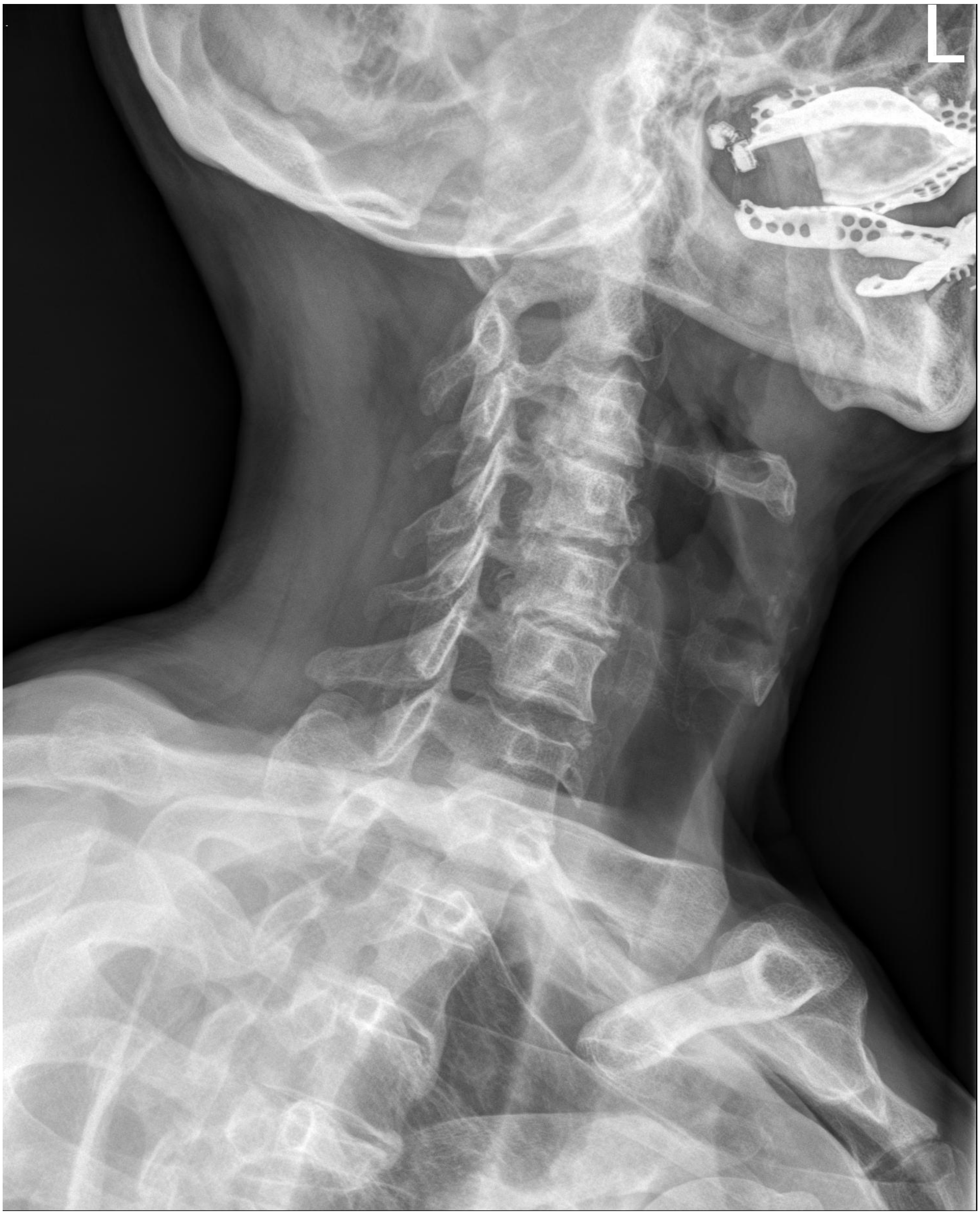

	C23	C34	C45	C56	C67	Total score
Endplate sclerosis	0	1	1	1	0	3
Disc space narrowing	0	1	1	2	0	4
Anterior osteophyte	0	1	1	2	1	5
Posterior osteophyte	0	1	1	1	0	3
Listhesis	0	0	0	0	0	0
Facet joint	0	0	0	0	0	0
Total score	0	4	4	6	1	15

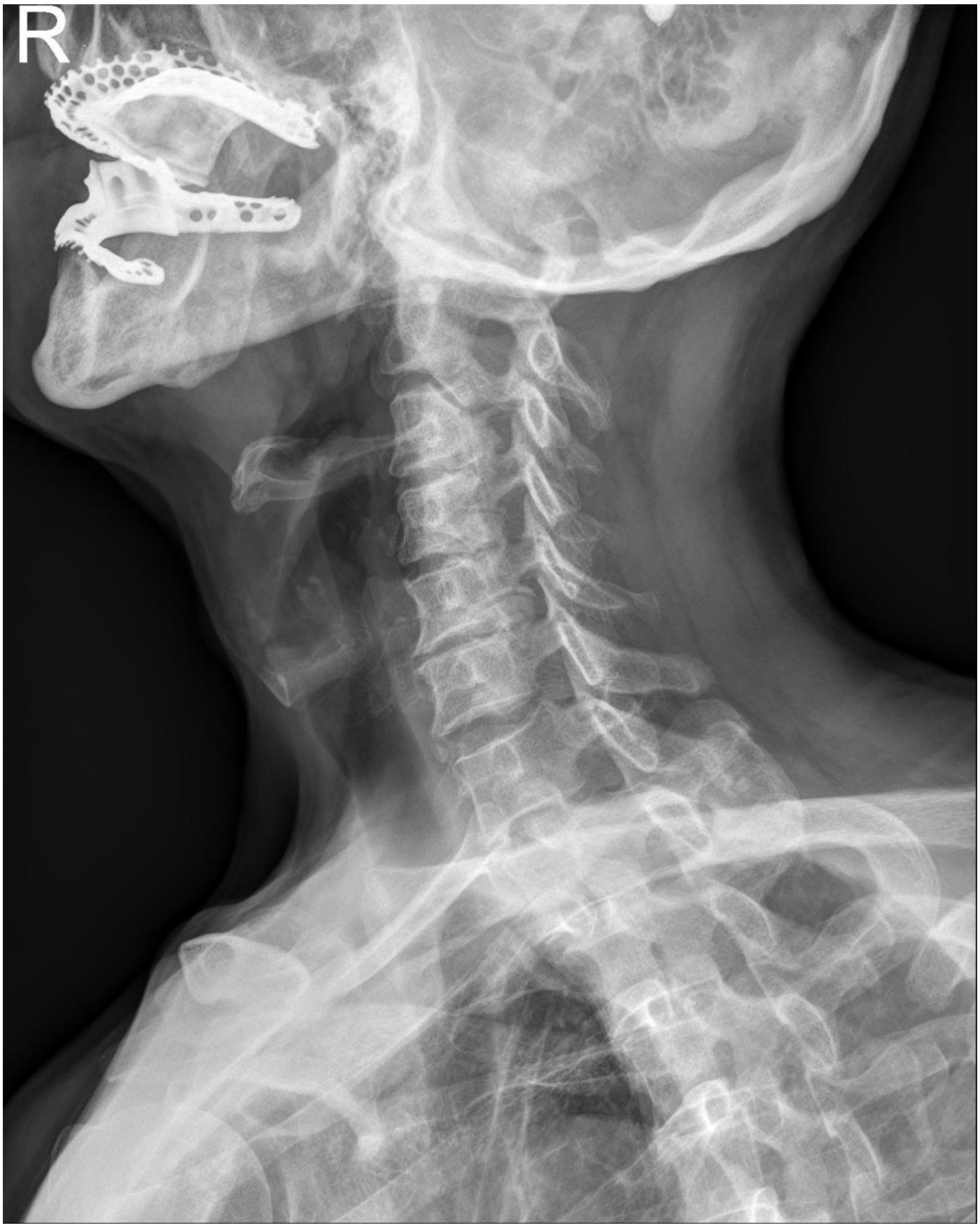

	C23	C34	C45	C56	C67	Total score
Endplate sclerosis	0	1	1	1	0	3
Disc space narrowing	0	1	1	2	0	4
Anterior osteophyte	0	1	1	2	1	5
Posterior osteophyte	0	1	1	1	0	3
Listhesis	0	0	0	0	0	0
Facet joint	0	0	0	0	0	0
Total score	0	4	4	6	1	15

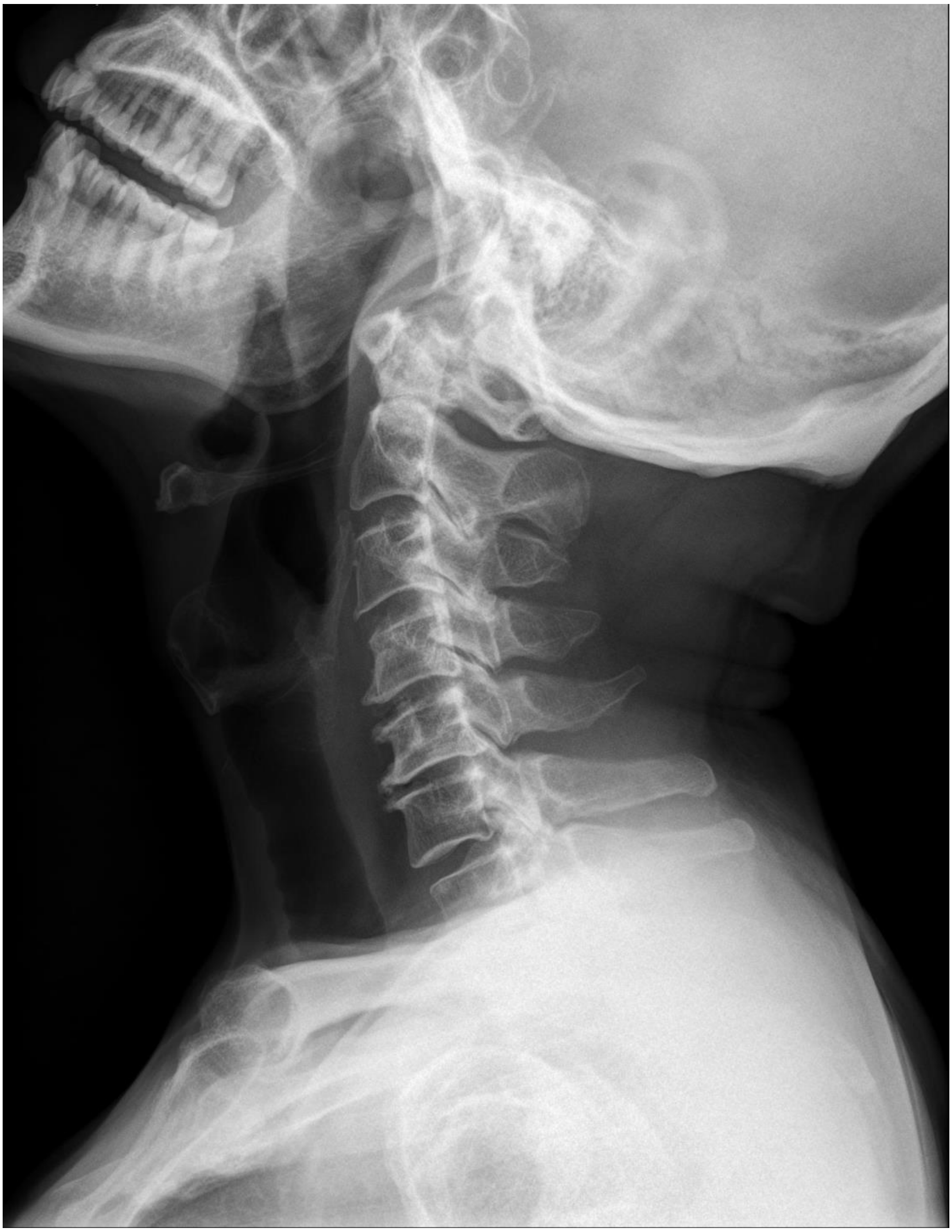

	C23	C34	C45	C56	C67	Total score
Endplate sclerosis	0	1	1	1	0	3
Disc space narrowing	0	1	1	2	0	4
Anterior osteophyte	0	1	1	2	1	5
Posterior osteophyte	0	1	1	1	0	3
Listhesis	0	0	0	0	0	0
Facet joint	0	0	0	0	0	0
Total score	0	4	4	6	1	15

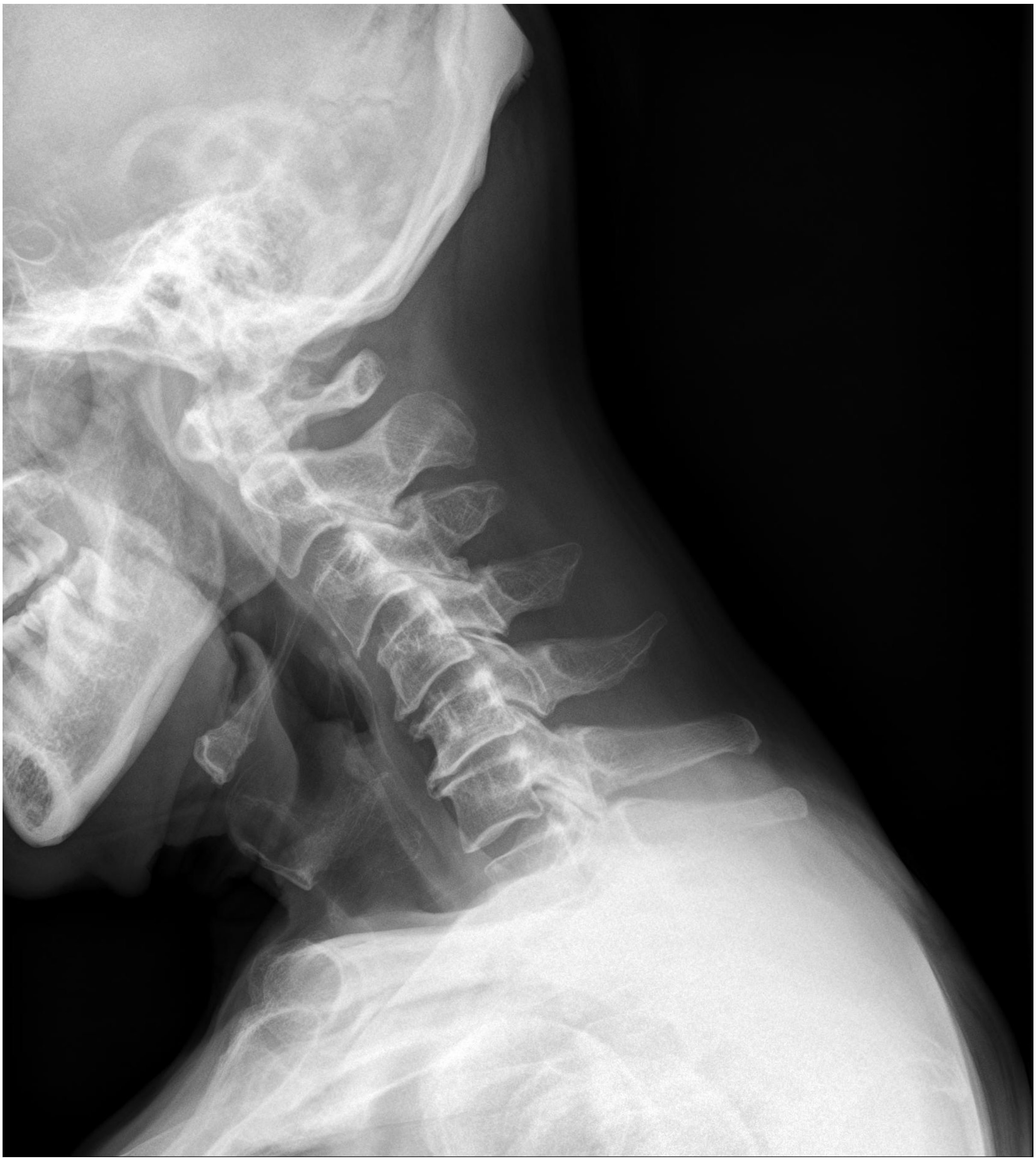

	C23	C34	C45	C56	C67	Total score
Endplate sclerosis	0	1	1	1	0	3
Disc space narrowing	0	1	1	2	0	4
Anterior osteophyte	0	1	1	2	1	5
Posterior osteophyte	0	1	1	1	0	3
Listhesis	0	0	0	0	0	0
Facet joint	0	0	0	0	0	0
Total score	0	4	4	6	1	15

Sample 6.

57/ Female

C2-7 SVA: 5.3 mm

C2-7 ARA: 23.3°

	C23	C34	C45	C56	C67	Total score
Endplate sclerosis	0	0	1	1	0	2
Disc space narrowing	0	0	0	1	0	1
Anterior osteophyte	0	0	1	0	0	1
Posterior osteophyte	0	0	1	1	1	3
Listhesis	0	0	1	1	0	2
Facet joint	0	0	0	0	1	1
Total score	0	0	4	4	2	10

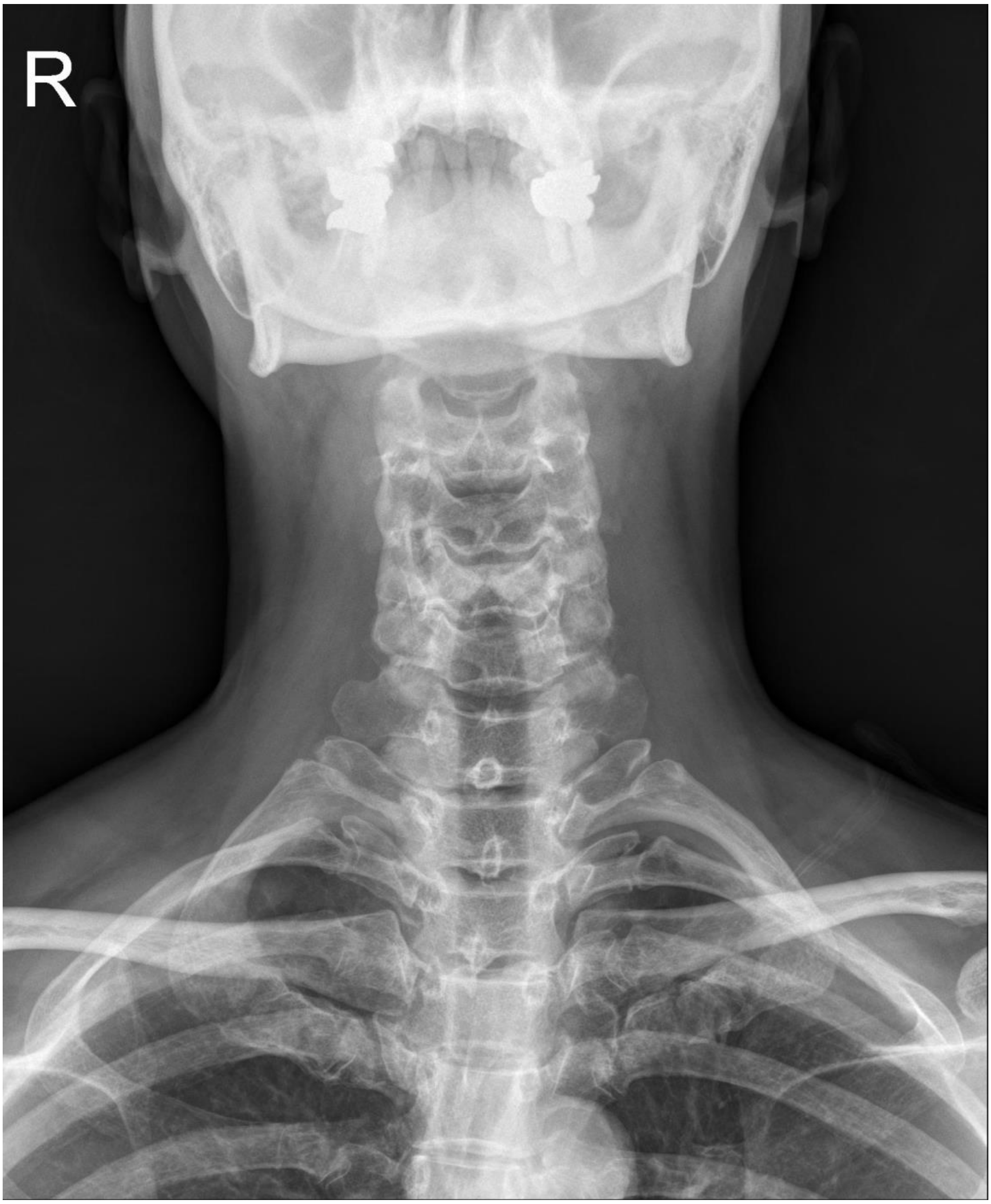

	C23	C34	C45	C56	C67	Total score
Endplate sclerosis	0	0	1	1	0	2
Disc space narrowing	0	0	0	1	0	1
Anterior osteophyte	0	0	1	0	0	1
Posterior osteophyte	0	0	1	1	1	3
Listhesis	0	0	1	1	0	2
Facet joint	0	0	0	0	1	1
Total score	0	0	4	4	2	10

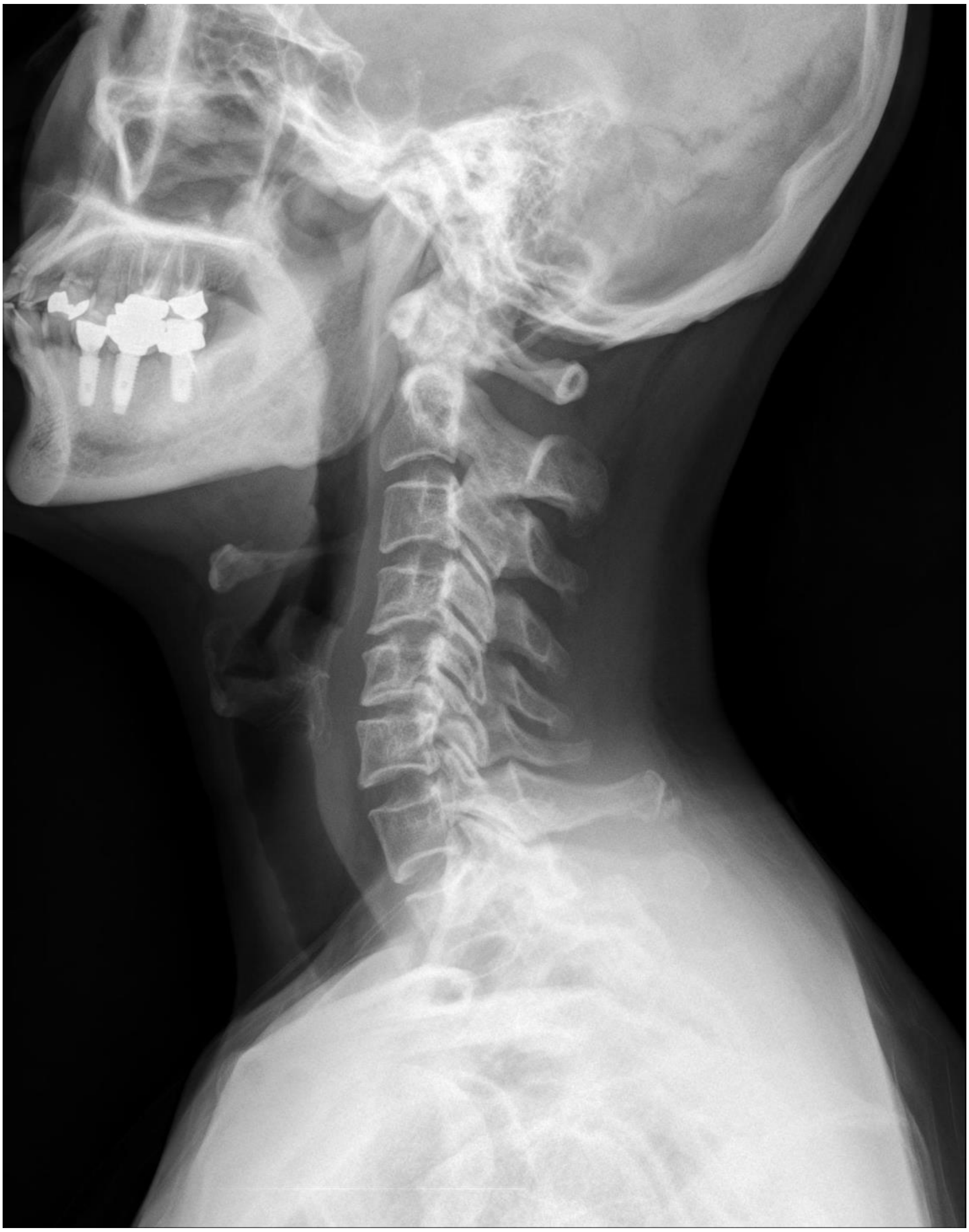

	C23	C34	C45	C56	C67	Total score
Endplate sclerosis	0	0	1	1	0	2
Disc space narrowing	0	0	0	1	0	1
Anterior osteophyte	0	0	1	0	0	1
Posterior osteophyte	0	0	1	1	1	3
Listhesis	0	0	1	1	0	2
Facet joint	0	0	0	0	1	1
Total score	0	0	4	4	2	10

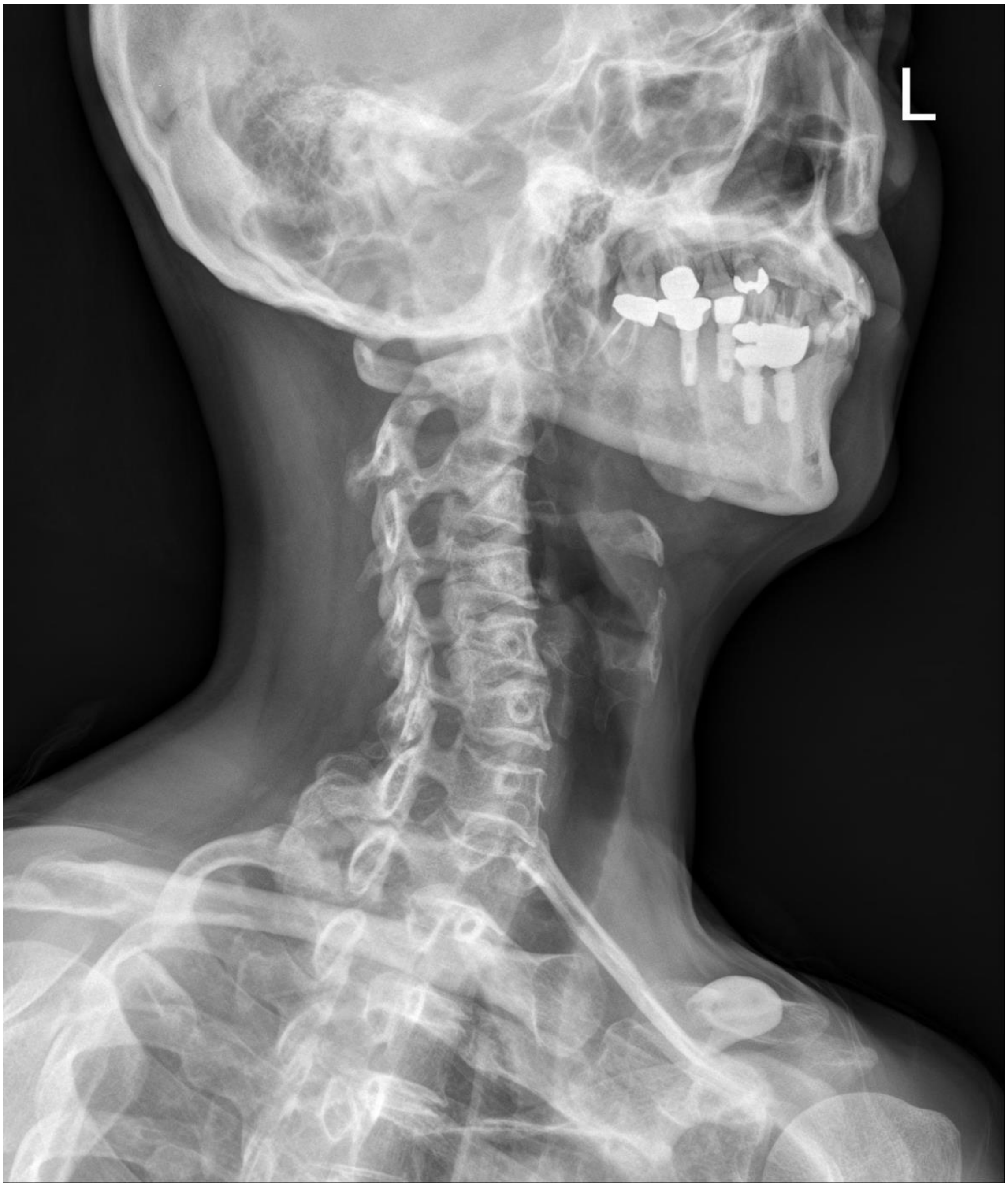

	C23	C34	C45	C56	C67	Total score
Endplate sclerosis	0	0	1	1	0	2
Disc space narrowing	0	0	0	1	0	1
Anterior osteophyte	0	0	1	0	0	1
Posterior osteophyte	0	0	1	1	1	3
Listhesis	0	0	1	1	0	2
Facet joint	0	0	0	0	1	1
Total score	0	0	4	4	2	10

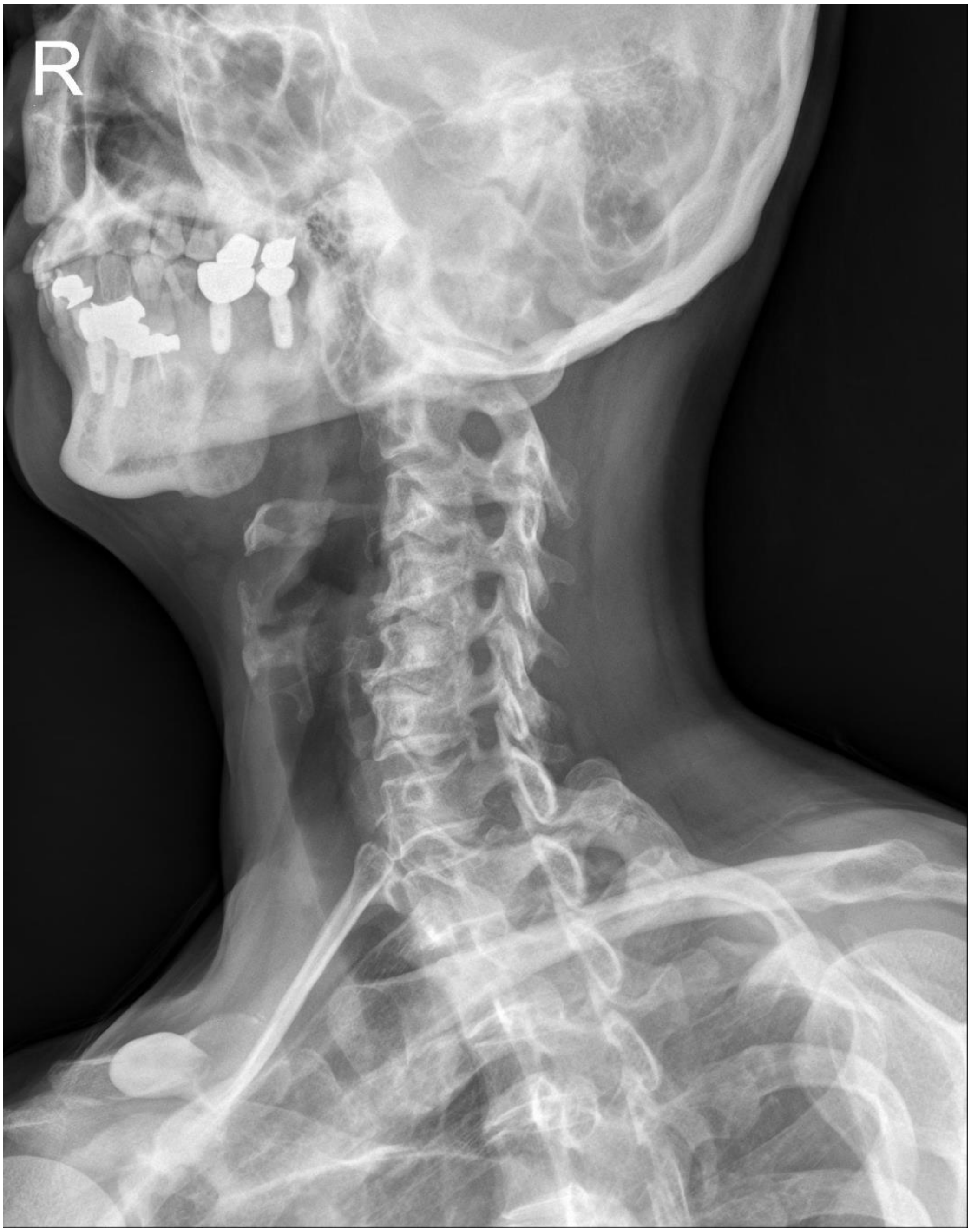

	C23	C34	C45	C56	C67	Total score
Endplate sclerosis	0	0	1	1	0	2
Disc space narrowing	0	0	0	1	0	1
Anterior osteophyte	0	0	1	0	0	1
Posterior osteophyte	0	0	1	1	1	3
Listhesis	0	0	1	1	0	2
Facet joint	0	0	0	0	1	1
Total score	0	0	4	4	2	10

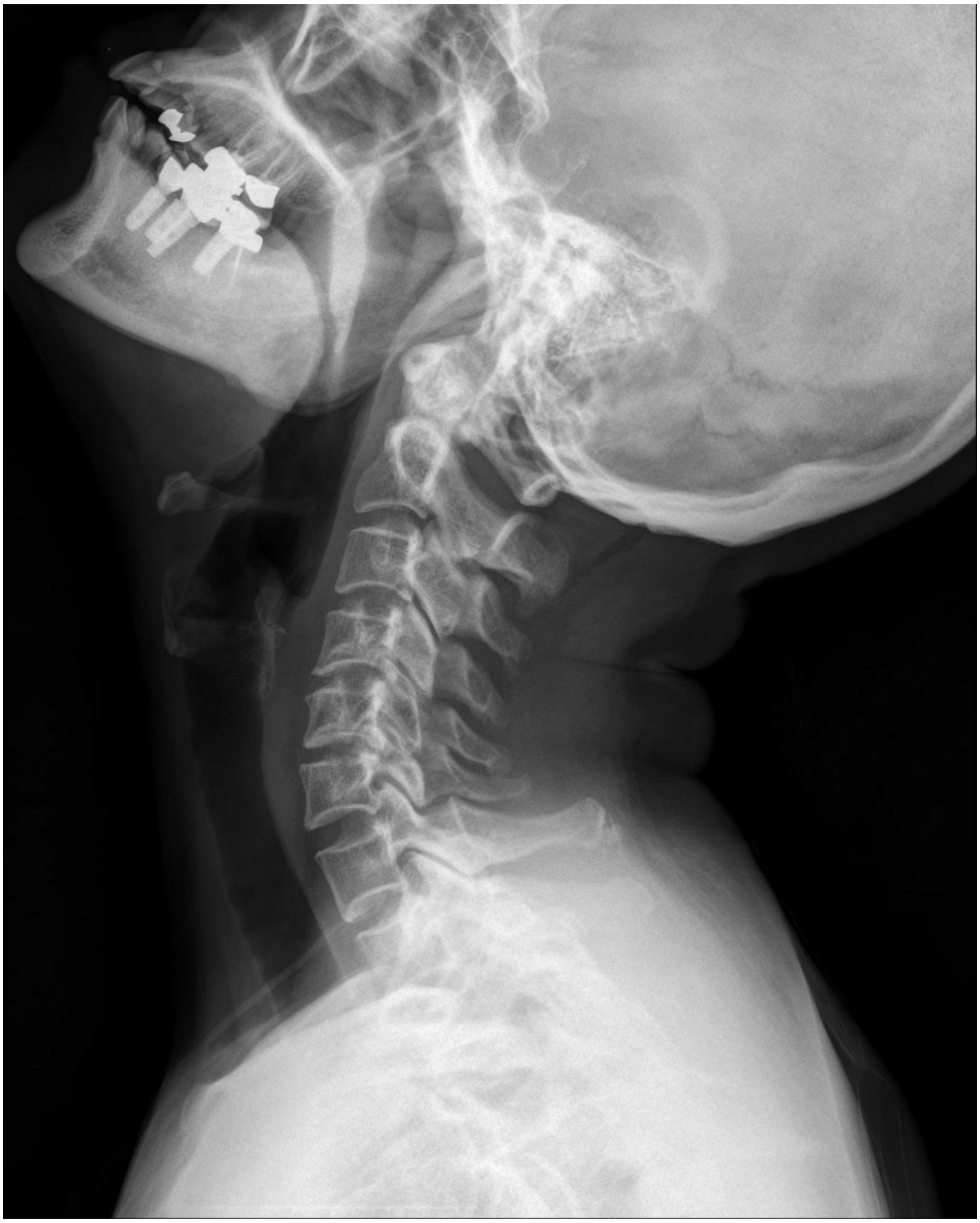

	C23	C34	C45	C56	C67	Total score
Endplate sclerosis	0	0	1	1	0	2
Disc space narrowing	0	0	0	1	0	1
Anterior osteophyte	0	0	1	0	0	1
Posterior osteophyte	0	0	1	1	1	3
Listhesis	0	0	1	1	0	2
Facet joint	0	0	0	0	1	1
Total score	0	0	4	4	2	10

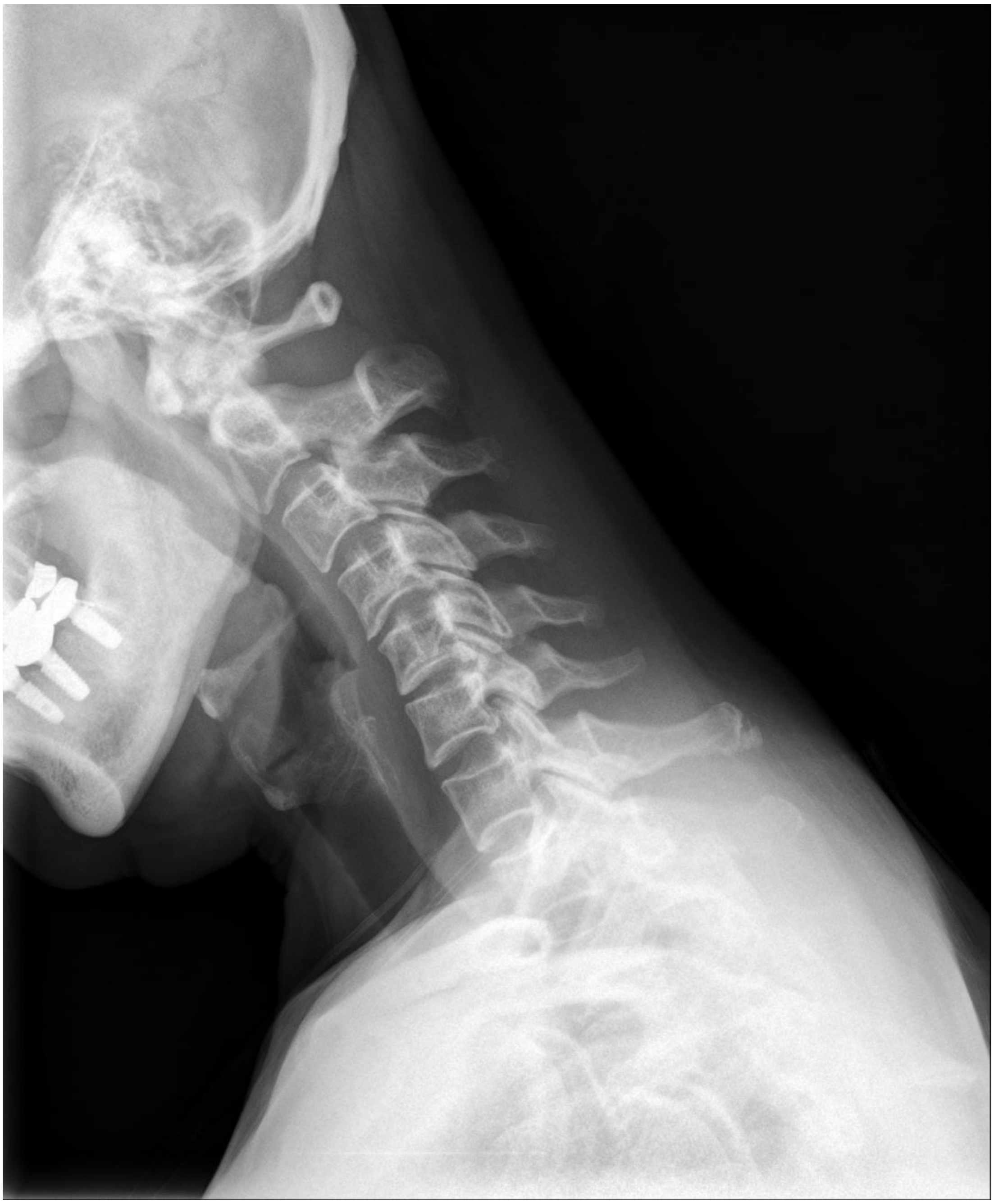

	C23	C34	C45	C56	C67	Total score
Endplate sclerosis	0	0	1	1	0	2
Disc space narrowing	0	0	0	1	0	1
Anterior osteophyte	0	0	1	0	0	1
Posterior osteophyte	0	0	1	1	1	3
Listhesis	0	0	1	1	0	2
Facet joint	0	0	0	0	1	1
Total score	0	0	4	4	2	10
