# Supplementary material for: Association between Sagittal Cervical Spinal Alignment and Degenerative Cervical Spondylosis: A Retrospective Study Using a New Scoring System
Source: J Clin Med. 2022 Mar 23;11(7):1772. doi: 10.3390/jcm11071772 (PMC8999493; doi:10.3390/jcm11071772)
Supplement: Supplementary file 1 [file jcm-11-01772-s001.zip › Table_S1.pdf]

Supplementary Table S1. Inter-rater correlation coefficients of the measured values.

	CC	*p*	95% CI
Endplate sclerosis	0.63	<0.001	0.59 to 0.67
Disc space narrowing	0.77	<0.001	0.74 to 0.80
Anterior osteophyte	0.86	<0.001	0.84 to 0.88
Posterior osteophyte	0.64	<0.001	0.60 to 0.68
Listhesis	0.61	<0.001	0.57 to 0.65
Facet joint	0.59	<0.001	0.54 to 0.63
Total	0.87	<0.001	0.85 to 0.88
C2-7 ARA	0.94	<0.001	0.93 to 0.94
C2-7 SVA	0.94	<0.001	0.93 to 0.94

CC: correlation coefficient, CI: confidence interval, ARA: absolute rotational angle, SVA: sagittal vertical axis
