# Supplementary material for: Association between Sagittal Cervical Spinal Alignment and Degenerative Cervical Spondylosis: A Retrospective Study Using a New Scoring System
Source: J Clin Med. 2022 Mar 23;11(7):1772. doi: 10.3390/jcm11071772 (PMC8999493; doi:10.3390/jcm11071772)
Supplement: Supplementary file 1 [file jcm-11-01772-s001.zip › Table_S2.pdf]

Supplementary Table S2. Intra-rater correlation coefficients of the measured values.

	Rater 1			Rater 2			Rater 3		
	CC	*p*	95% CI	CC	*p*	95% CI	CC	*p*	95% CI
Endplate sclerosis	0.88	<0.001	0.77 to 0.94	0.85	<0.001	0.71 to 0.92	0.78	<0.001	0.59 to 0.89
Disc space narrowing	0.86	<0.001	0.73 to 0.93	0.94	<0.001	0.88 to 0.97	0.76	<0.001	0.57 to 0.88
Anterior osteophyte	0.91	<0.001	0.83 to 0.96	0.99	<0.001	0.98 to 0.99	0.90	<0.001	0.82 to 0.95
Posterior osteophyte	0.81	<0.001	0.66 to 0.90	0.79	<0.001	0.62 to 0.89	0.82	<0.001	0.66 to 0.91
Listhesis	0.73	<0.001	0.43 to 0.87	0.93	<0.001	0.85 to 0.96	0.73	<0.001	0.52 to 0.86
Facet joint	0.69	<0.001	0.34 to 0.85	0.80	<0.001	0.62 to 0.90	0.85	<0.001	0.72 to 0.93
Total score	0.92	<0.001	0.84 to 0.96	0.94	<0.001	0.89 to 0.97	0.92	<0.001	0.85 to 0.96
C2-7 ARA	0.96	<0.001	0.90 to 0.98	0.94	<0.001	0.87 to 0.97	0.95	<0.001	0.90 to 0.98
C2-7 SVA	1.00	<0.001	1.00 to 1.00	1.00	<0.001	1.00 to 1.00	0.99	<0.001	0.98 to 1.00

CC: correlation coefficient, CI: confidence interval, ARA: absolute rotational angle, SVA: sagittal vertical axis
