# Supplementary material for: Association between Sagittal Cervical Spinal Alignment and Degenerative Cervical Spondylosis: A Retrospective Study Using a New Scoring System
Source: J Clin Med. 2022 Mar 23;11(7):1772. doi: 10.3390/jcm11071772 (PMC8999493; doi:10.3390/jcm11071772)
Supplement: Supplementary file 1 [file jcm-11-01772-s001.zip › Table_S3.pdf]

Supplementary Table S3. Description of the causal mediation effect model.

Linear model without mediator: DCS score = Sex + Age + C2-7 SVA				
	Estimate	std	t-value	p-value
(Intercept)	-8.943	0.969	-9.226	0.000
Sex (male)	3.381	0.485	6.968	0.000
Age	0.308	0.016	19.697	0.000
C2-7 SVA	0.016	0.023	0.702	0.483
Linear model for mediator: C2-7 ARA = Sex + Age + C2-7 SVA				
	Estimate	std	t-value	p-value
(Intercept)	-8.730	1.928	-4.528	0.000
Sex (male)	-0.862	0.965	-0.893	0.372
Age	-0.229	0.031	-7.373	0.000
C2-7 SVA	0.299	0.046	6.446	0.000
Mediation model: DCS score = Sex + Age + C2-7 SVA + C2-7 ARA				
	Estimate	95% CI lower	95% CI upper	p-value
ACME	-0.039	-0.056	-0.030	0.000
ADE	0.349	0.319	0.380	0.000
Total effect	0.308	0.280	0.340	0.000
Proportion of mediation	-0.125	-0.188	-0.080	0.000

DCS: degenerative cervical spondylosis, ARA: absolute rotational angle, SVA: sagittal vertical axis, ACME: average causal mediation effect, ADE: average direct effect, CI: confidence interval.
